# Context, mechanisms and outcomes of dementia special care units: An initial programme theory based on realist methodology

**DOI:** 10.1371/journal.pone.0259496

**Published:** 2021-11-16

**Authors:** Rebecca Palm, Anne Fahsold, Martina Roes, Bernhard Holle

**Affiliations:** 1 Faculty of Health, Witten/Herdecke University, School of Nursing Science, Witten, Germany; 2 German Center for Neurodegenerative Diseases, Witten, Germany; University of Alberta, CANADA

## Abstract

**Background:**

Dementia special care units represent a widely implemented care model in nursing homes. Their benefits must be thoroughly evaluated given the risk of exclusion and stigma. The aim of this study is to present an initial programme theory that follows the principles of realist methodology. The theory development was guided by the question of the mechanisms at play in the context of dementia special care units to produce or influence outcomes of interest in people with dementia.

**Methods:**

The initial programme theory is based on qualitative interviews with dementia special care stakeholders in Germany and a realist review of complex interventions in dementia special care units. The interviews were analysed using content analysis techniques. For the realist review, a systematic literature search was conducted in four scientific databases; studies were appraised for quality and relevance. All data were analysed independently by two researchers. A realist informed logic model was developed, and context-mechanism-outcome (CMO) configurations were described.

**Results:**

We reviewed 16 empirical studies and interviewed 16 stakeholders. In the interviews, contextual factors at the system, organisation and individual levels that influence the provision of care in dementia special care units were discussed. The interviewees described the following four interventions typical of dementia special care units: adaptation to the environment, family and public involvement, provision of activities and behaviour management. With exception of family and public involvement, these interventions were the focus of the reviewed studies. The outcomes of interest of stakeholders include responsive behaviour and quality of life, which were also investigated in the empirical studies. By combining data from interviews and a realist review, we framed three CMO configurations relevant to environment, activity, and behaviour management.

**Discussion:**

As important contextual factors of dementia special care units, we discuss the transparency of policies to regulate dementia care, segregation and admission policies, purposeful recruitment and education of staff and a good fit between residents and their environment.

## Introduction

Dementia is a non-communicable neurodegenerative disease that peaks in old age [1]. Due to demographic changes, we expect the number of people with dementia to increase worldwide without any prospects for preventive measures, cures or treatments [2]. A main symptom of dementia is cognitive decline, which manifests as memory and function loss, behavioural change (is named responsive behaviour below) and increasing dependency on others [3]. During the late stages of the disease, moving into a nursing home is difficult to avoid, which is why in many countries, most nursing home residents live with dementia [4,5].

A major issue regarding the provision of nursing home care for people with dementia is the question of whether they should live in a dementia special care unit. Despite the absence of clear evidence of effectiveness [6], dementia special care units have gained wide acceptance in practice such that numerous different dementia special care units currently exist worldwide. These units, which are called Alzheimer’s special care units or small-scale living units, vary in size, staff, environment, care practices and quality of care. In general, dementia special care units share the idea that care for people with dementia can be better provided in a special environment with staff that is better trained to care for people with dementia. In general, these units are available only for people with dementia. Therefore, an environment specially designed for people with dementia and staff specially trained in certain care practices for people with dementia (e.g., dementia care mapping) are common characteristics of dementia special care units [7,8].

A common definition of their standards or regulations is lacking in many countries, such as Germany. We estimate that in Germany, 30% of nursing homes have implemented a dementia special care unit, but valid and actual data are still lacking [9]. Furthermore, how many of the nearly 16,000 nursing home beds in Germany are a part of a dementia special care unit is unknown. In the U.S., current data show that 4% of 74,000 nursing home beds are in Alzheimer’s special care units [1].

The question of whether dementia special care units enhance the quality of life or foster social exclusion and stigma has ongoing serious implications for practice, policy and research [2]. Despite a large body of knowledge regarding dementia special care units, clear recommendations in global action plans (https://apps.who.int/iris/bitstream/handle/10665/259615/9789241513487-eng.pdf;jsessionid=259CCEB6BF22D80753ECEE02881B49C3?sequence=1) and the German national dementia strategy are missing (https://www.nationale-demenzstrategie.de/die-strategie). The possible reasons why recommendations or directional decisions are still lacking may be contradictory research results and the many methodological challenges that empirical studies face. Previous studies comparing dementia special care units with traditional units mostly failed to show clear evidence of benefits; however, these studies could demonstrate advantages only in certain measures [10–14]. A recent study using claims data from the U.S. clearly shows that nursing homes with a dementia special care unit provide a higher quality of care as measured by different indicators [15]. These studies all have several methodological problems; one such problem is a missing definition of dementia special care units. Because there are no uniform standards and regulations, nursing homes provide very different models of dementia special care units. Furthermore, nursing homes without dementia special care units also offer elements of dementia special care because they accommodate many residents with dementia. Studies adopting a control group design that define their study groups based on the distinguishing feature of segregation are prone to bias because units differ from one another, and comparison groups may share elements in common with dementia special care units. While the problem is not new, it remains unsolved [15–17]. Another major problem is that residents who live in dementia special care units often have different characteristics than residents who live in other types of care units, which applies to not only observable but also non-observable characteristics, e.g., disease trajectories and past experiences with care providers, making it difficult to adjust for these differences [14,15].

Another reason why it is difficult to interpret the results of empirical research concerning dementia special care units is the question of which outcome is the most important and promising. Many investigators questioned whether dementia special care units reduce unpleasant outcomes, such as functional or cognitive decline or the severe responsive behaviour, and whether dementia special care units are able to enhance the quality of life [6,10,13]. Given the disillusioning results, discussing whether the investigated outcomes or the assumed mechanisms underlying the outcomes are grounded on a sound theoretical basis or whether other mechanisms that researchers have not previously considered influence the outcomes is warranted.

The recommendations regarding the effectiveness of care interventions for people with dementia are clearer. Evidence supporting the use of person-centred psychosocial interventions is increasing; thus, care providers might be expected to implement such interventions [18]. The authors of a Cochrane Review of dementia special care units also emphasised that it might be more important to implement best practices than provide a special environment [6]. From an implementation perspective, this conclusion seems rather brief because the effectiveness of an intervention always needs to be evaluated in the context of where it is implemented. Hence, we cannot consider care interventions and the context separately, leading to the question of whether the effectiveness of person-centred psychosocial interventions for people with dementia depends on the context in which these interventions are implemented, and if so, do dementia special care units contribute to their effectiveness?

Considering these issues, we adopt an alternative evaluation approach that aims not to answer the question of whether dementia special care units are beneficial but rather to investigate the mechanisms at play in the context of dementia special care units to produce or influence outcomes of interest in people with dementia. As an evaluation approach, we follow the realist evaluation proposed by Pawson & Tilley [19].

## Methodological background

### Realist methodology and the development of initial programme theories

The realist methodology is a relatively novel approach to evaluating complex interventions in health care. Developed in the 1990s by Pawson & Tilly in the UK [19], the realist methodology is gaining increasing attention in nursing research [20] and other health care research fields [21]. The realist methodology aims to uncover the mechanisms that underlie interventions and are responsible for a change in outcomes. In the understanding of Pawson & Tilley, a complex intervention is effective in changing outcomes only if it releases the underlying mechanisms that are needed to make the intervention work. To be released, mechanisms need certain contextual conditions, and if such contextual conditions do not exist, even the most powerful intervention will not be effective. In summary, realists argue that it is not the intervention but the underlying mechanisms that work in a certain context.

The central tenet of a realist evaluation is programme theory, which formulates an assumption regarding how the context, mechanism and outcomes relate to each other [19]. Programme theory explains why an intervention works, in which context and for whom. Hence, the study objectives of realist evaluations are not interventions or programmes but programme theory. Programme theories are operationalised as context-mechanism-outcome (CMO) configurations that display their inter-relatedness.

The development of an initial programme theory is the starting point of a realist evaluation. By the nature of the iterative research process, an initial programme theory is subject to continual change until it can be presented as a refined programme theory [22]. To improve the methodological clarity, coherence and transparency, we describe this development process and define the central concepts and terms.

### Definition of terms

We define *context* as the features of the situation in which the intervention is implemented that interact with its operation [23]. We explicitly note that context is not equal to *setting*. With the description of context, we seek to identify the features of dementia special care units in nursing homes that differ between organisations that affect how dementia-specific interventions work.

We understand *dementia-specific interventions* as complex interventions that seek to achieve a change in the outcomes of residents with dementia by administering the intervention either to a resident or others (e.g., staff members) who are expected to change their behaviour to improve the residents’ *outcomes*. Hence, we expect *interventions* to influence a resident’s *outcomes* indirectly if the intervention targets intermediate outcomes, such as staff behaviour. Following Clark’s view regarding complexity [24], we consider that *complex interventions* work in a non-linear, non-static way without clear, direct and consistent links between the components of the interventions and (*intermediate) outcomes*. We define *complex interventions*, according to Clark [24], as interventions formed of parts that can be material, human, theoretical, social or procedural.

We understand *mechanisms* as the underlying causal processes that lead to intended or unintended changes in *outcomes* [19]. Mechanisms are an individual’s interpretation of or reaction (reasoning) to a resource provided by an intervention. The distinction between intervention resources and reasoning is used to operationalise mechanism components and distinguish whether data contribute to programme theory as a part of the context or part of a mechanism [25]. Programme mechanisms are generally not observable or measurable, but they are nonetheless real [26]. Such mechanisms need to be abstracted and deduced from patterns that become evident in reality.

Regarding *outcome*, we followed the concept described by Paterson et al. [27], who define outcomes as health-related changes that result from an interaction among an intervention, process and context over time. We broaden this definition with respect to psychosocial outcomes, which are of great relevance for people with dementia. *Outcomes* may be independent of or dependent on residents’ awareness. We understand *intermediate outcomes* as necessary to reach a change in outcomes and, therefore, as preceding the outcome. *Intermediate outcomes* can be qualitative or quantitative in nature and encompass participation, fidelity, barriers and facilitators of intervention implementation [28].

## Aim and research questions

Our aim is to present an initial programme theory explaining the mechanisms at play in the context of dementia special care units to produce or influence outcomes of interest in people with dementia. Here, the initial programme theory includes different forms of dementia special care units (e.g. Alzheimer’s Special Care Units and small-scale living units for people with dementia). While explaining the mechanism, we focus on the complex interventions that are generally provided in dementia special care units. The initial theory focuses on residents’ outcomes that stakeholders consider essential. To achieve the study aim, we applied the following three-step approach:

I Step: Description of the prevailing contextual factors of dementia special care units, dementia-specific interventions that have been implemented in dementia special care units and outcomes in the context of dementia special care units that are of relevance for stakeholders.

Regarding the description, we focus on the following research questions:

*Which contextual factors are characteristic of dementia special care units in Germany from the point of view of stakeholders*?
*Which dementia-specific interventions delivered in these units are considered characteristic of dementia special care units from the viewpoint of stakeholders*?
*Which outcomes of dementia special care units are relevant*, *and what changes in outcomes are intended when people with dementia are admitted*?

II Step: Development of a realist informed iterative logic model that visualises the context, mechanism resources, mechanism reasoning, intermediate outcomes and outcomes (theory of action).
III Step: Development of CMO configurations that explain why residents’ outcomes change (or why they do not change) (theory of change).

For the development of the CMO configurations, we focused on the following research question: *what aspect of the context is relevant for achieving the desired outcomes by releasing the underlying mechanisms of dementia-specific interventions*?

## Methods

### Process of theory development

**Fig 1:**
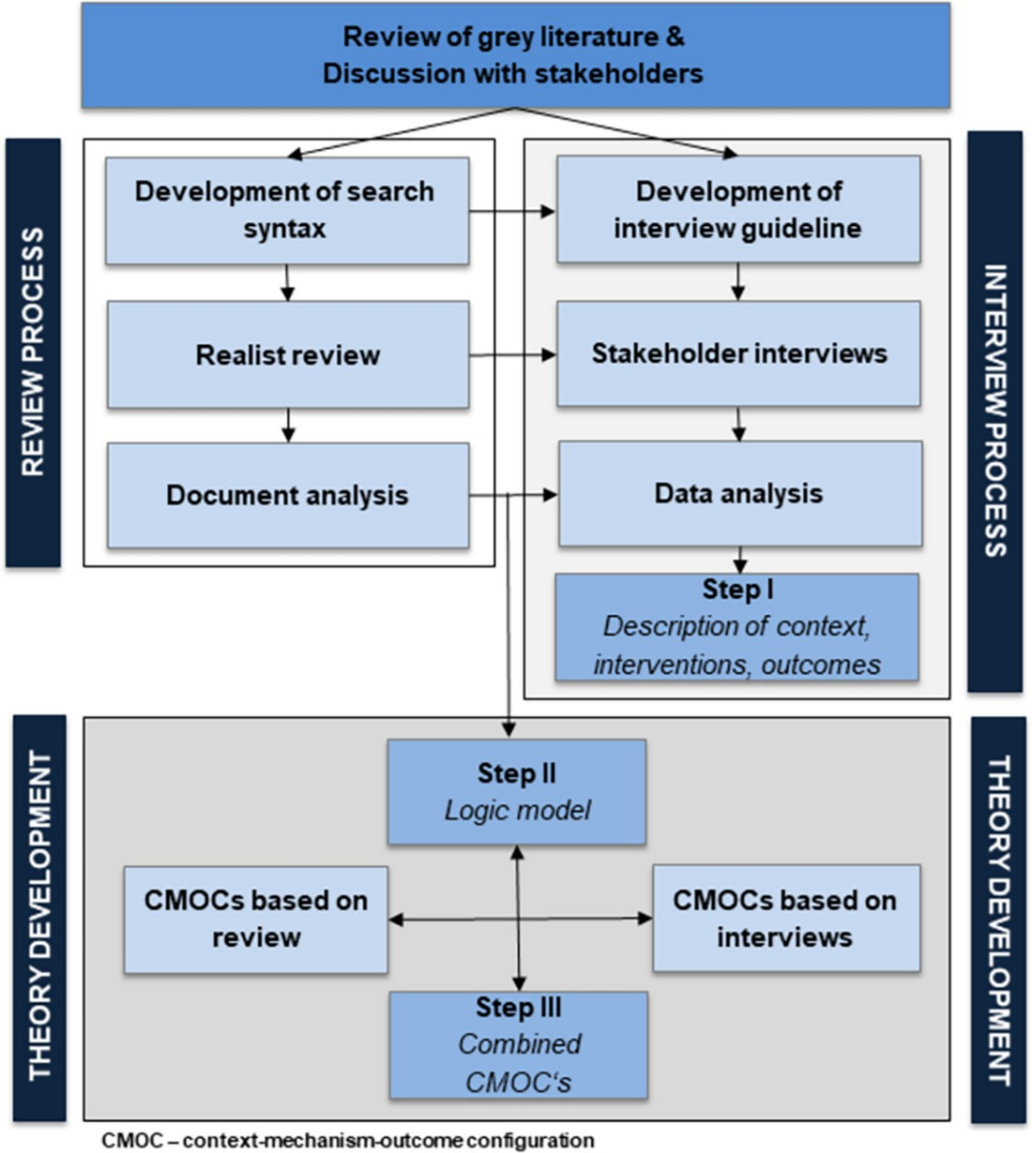
Process of theory development (self-developed figure).

The results of qualitative stakeholder interviews and the findings of empirical studies that investigated complex interventions in dementia special care units are the basis of the development of the initial programme theory. We started the process by reviewing the grey literature to map the concept of dementia special care units and how they are operated in the real world. The review was complemented with discussions with dementia special care unit stakeholders who also recommended further grey literature. Stakeholders of dementia special care units were recruited from the professional network of the first author. Information from the grey literature and the stakeholders formed the basis of the interview guideline, which the stakeholders reviewed and supplemented. The topics in the interview guideline formed the basis of the search syntax for the systematic review. The theory development process is shown in Fig 1.

#### Step 1—Description of context, dementia-specific interventions and outcomes

We used stakeholder interviews to describe the prevailing context of dementia special care units, dementia-specific interventions and their application in dementia special care units and outcomes relevant for stakeholders in German nursing homes.

#### Step 2—Development of a realist informed logic model (theory of action)

For the theory development, we combined the logic model approach of depicting theoretical models of complete interventions [28] with Pawson & Tilley’s logic of inquiry of the realist evaluation [19]. We combined the approaches to compensate for the shortcomings of each approach [29]. Logic models are typically designed to provide a graphic description of the system in which an intervention is implemented and how dementia-specific interventions are embedded in this context. The layout of the logic model was inspired by the templates of logic models provided by Rohwer et al. [28]. We developed our logic model via an iterative approach; thus, we changed the logic model during the data analysis process. The first version of the logic model was based on the knowledge we obtained from analysing the stakeholder interviews. We continuously adapted our logic model by integrating findings from the literature into the model. We also included mechanisms in the logic model, which is a concept inherent in the realist methodology. The final logic model visualises the *context*, *inputs*, *interventions*, *mechanism*, *intermediate outcomes* and *outcomes*. We consider the logic model the starting point of our programme theory.

#### Step 3—Development of the CMO configurations (theory of change)

The theory of change is based on the CMO configurations derived from the interviews and the included studies. We (RP and AF) developed the CMO configurations independently of each other and then compared them. We discussed and harmonised the incongruent CMO configurations. For each study, the link among the context, mechanism and outcome was described as a part of the quality appraisal by formulating *“if…then…because”* statements. We combined CMO configurations from different studies and interviews that had the same focus and summarised a single CMO configuration as a possible hypothesis explaining why the context of dementia-specific interventions has an impact on the outcomes.

### Stakeholder interviews

The study was reviewed by the ethics committee of the German Society for Nursing Science. The approval number is 16–015 (31.05.2016). All participants gave their written consent.

Interviews were conducted with different dementia special care unit stakeholders (nursing home providers, health insurance representatives and local health/social authorities) by the first author (RP) in 2017. We selected the interviewees based on their professional experience leading a dementia special care unit since we expected that these interviewees could provide detailed information regarding the context. The aim was to include people who can provide detailed information regarding the context of dementia special care and dementia special care units. The participants were recruited from the professional network of the authors, who acted as gatekeepers, or identified based on their expertise. The topics included in the interview guide covered the characteristics of the dementia special care unit for people with dementia, target group, goals of the dementia special care unit, reason for the implementation of the dementia special care unit, regulations and financing and problems experienced in the dementia special care unit. The nature of the interview questions was descriptive, and we aimed to develop theoretical assumptions based on these interviews. All participants were invited to the interview by mail and phone. The interviews were conducted at the work settings of the interviewees. The interviews were recorded and transcribed verbatim by a professional agency. The transcripts were not returned to or discussed with the participants. The first and second authors analysed the data using MAXQDA 2018 [30]. Initially, these authors read and re-read the transcripts to become familiar with the material. Then, these authors extracted the data following the principles of a qualitative content analysis according to Mayring using a deductive-inductive approach [31]. Therefore, deductively formed categories based on the interview guide constituted the initial coding scheme. During the analysis of the individual transcripts, inductive categories evolved from the interview transcripts and were included in the schema. This step of the analysis was carried out independently by the authors RP and AF. In the second step, a common version of their coding schemes was created (see S1 Table). The coding schemes contained in this version were discussed, and the topics of interest were summarised for further data synthesis. The coding process was continued until all text was consistently categorised. The content categories were then allocated to context, intervention and outcomes. We merged the findings of the realist review and the interviews. Based on this analysis, we categorised the interventions and outcomes derived from the findings in the review and allowed a common assignment.

### Realist review

We conducted a realist review following the principles outlined for this method [32,33]. A systematic literature search was conducted using four databases (PubMed, CINAHL, PsycINFO, and Scopus). The search was performed in February and March 2018 and was restricted to the German and English languages and publication dates from 2008 to January 2018. The search terms were special care units (and synonyms), dementia (and synonyms) and long-term care (and synonyms) in titles and abstracts. Additionally, indexing terms (i.e., MeSH terms) were used if possible. The search strategies were adapted to the different databases. The search syntax can be found in the supplementary material (S2 Table). The included literature was related to care for people with dementia in special care units in nursing homes. Because many studies did not clearly define special care units and it was not always obvious if a special care unit was synonymous with a segregated care unit, we also included studies that defined units as dementia special care units. We applied this broad definition of dementia special care units because they are operated differently internationally. All study designs, topics, disciplinary perspectives and research questions were included if the focus was on dementia special care units. The inclusion and exclusion decisions were made via the following two steps: first after screening the title and abstract of a reference and second after reading the full text. The exclusion criteria are shown in Table 1. For each text, we completed a quality appraisal based on realist principles that assessed the relevance and rigour of the included studies (S1 File). We judged the relevance as high if the research and the research questions were closely aligned with the focus points of the review and there was a substantial description of the *context*, *mechanism* and *outcomes* that informed the review. The descriptions of the “moderate” and “low” categories can be found in the quality appraisal form. A study was included if we judged it to have at least moderate relevance for the review. We excluded studies with low relevance. Studies with low rigour but moderate or high relevance were included. Following the recommendations for conducting a realist review [33], we developed CMO configurations based on each study. Finally, the CMO configurations developed for each study were summarised into three superordinate CMO configurations. The literature selection, quality appraisal and development of CMO configurations were conducted by the first and second authors independently and discussed in the case of incongruity until consensus was reached.

**Table 1:** Exclusion criteria for the literature screening.

Title/Abstract screening	Full-text screening
No abstract is available	No full text is available
Publication format is inadequate (e.g., commentary, letter to the editor, or study protocol)	Outcomes focus exclusively on staff or relatives and not on residents
Publication does NOT report an empirical study (e.g., book section) or reports a literature overview that is not based on a systematic literature search	Interventions provided in dementia special care units are not clearly described or are not the focus of the study
	Interventions are not defined a priori as typical for dementia special care units, e.g., end-of-life care
	Setting is not a nursing home
	Intervention does not fit the logic model (e.g., provision of end-of-life care on DSCUs)

## Results

### Description of the interview participants

We conducted interviews with 16 participants working in 11 institutions. The stakeholders represented the perspective of nursing home providers, nursing home care regulatory authorities and cost bearers. The characteristics of the stakeholders are shown in Table 2.

**Table 2:** Characteristics of the interview participants.

ID	Job position	Years of work experience^1^	Institution
SH 01	Head of the NH	7	Nursing home with 165 beds, including 15 beds in a dementia special care unit
SH 02*	Head of the NH	7	Nursing home with 194 beds, including 32 beds in a dementia special care unit
SH 03*	Nursing director	1	
SH 04*	Head of the NH	11	Nursing home with 120 beds, including 14 beds in a dementia special care unit
SH 05*	Head of the DSCU	9	
SH 06	Head of the NH	11	Nursing home with 250 beds, including 112 beds in a dementia special care unit
SH 07	Nursing director	5	
SH 08*	Head of the NH	Unknown	Nursing home with 235 beds, including 32 beds in a dementia special care unit
SH 09*	Head of the DSCU	8	
SH 10	Nursing director	1	Nursing home with 42 beds, including 32 beds in a dementia special care unit
SH 11*	Head of the NH	8	Nursing home with 150 beds, including 32 beds in a dementia special care unit
SH 12*	Nursing director	13	
SH 13	Head of the DSCU	25	Health authority and self-governance
SH 14	Inspector	2	Health authority and nursing home control
SH 15	Head of the DSCU	2	Statutory long-term care insurance
SH 16	Skilled employee	1	Senate department and division care structures

^1^ In the current position.

*Stakeholders (SH) SH 02 and 03, 04 and 05, 06 and 07, 08 and 09, and 11 and 12 worked in the same institution and were interviewed together.

### Description of the context, dementia-specific interventions and outcomes from the viewpoint of the interviewees

#### Context—System (resources and regulation)

The economic resources of a nursing home depend on what the provider negotiated with the cost bearers (nursing home prices and costs). Higher staff ratios are possible only if a separate contract has been negotiated. If a nursing home aims to conclude a separate contract with the cost bearers, this can be achieved only if the regulations of the municipality allow it and the institution meets the necessary criteria.

The stakeholders commented that the characteristics of special care for people with dementia are often not published and available in all federal states and are binding in only a few states. Nevertheless, not all interviewees considered a legal specification of dementia special care units sensible.

#### Context—System (segregation and admission policies)

The ethical context has strong implications for daily practice as segregation implies the selection of residents who are to be admitted to these units. Admission and transfer policies are not addressed in the same way in all nursing homes. In some units, the residents are transferred when they no longer meet the admission criteria, whereas in other units, the residents can stay until their death. Transfer causes problems in daily life because residents and their relatives become accustomed to the physical and social environment of a unit and do not want to leave or have their relatives leave.

Another aspect considered a characteristic of dementia special care units is that residents are expected to participate in the provided activities. If residents can no longer participate because the disease has progressed too far, they are transferred from some units to another unit. However, transferring residents to receive end-of-life care was deemed unacceptable by the interviewees.

The interviewees all supported a special form of care offered in segregated nursing home areas if such units target people in a defined group, e.g., people with very severe dementia and severe responsive behaviour.

Segregation, however, was not unreservedly advocated by the interviewees. Furthermore, some participants addressed the contradiction posed by the concepts of segregation to the concepts of inclusion, with the latter being demanded on a political level. The interviewees emphasised that the aim of nursing home care should be to maintain normality and that this can be achieved more easily if people with different impairments live together and support each other. However, the interviewees also noted that a normal everyday life is not possible when people show severe responsive behaviour and are not adequately cared for. These special needs require an environment in which these needs can be met. A specialised nursing home is necessary as society is not yet ready to meet such needs differently.

#### Context—Organisation (staff)

The professionals who work in dementia special care units are considered their centrepiece. The most important elements of dementia special care units are careful staff selection, human resource development, the attitudes of staff members and staff-to-resident ratios. The interviewees reported that staff members must decide whether they want to work in a dementia special care unit, and superiors must carefully consider whether someone is suitable for a dementia special care unit.

Human resource development aims to develop the competence of nurses and care staff. Each nursing home included in our study had a special qualification training course that employees working with dementia patients had to complete. The qualification concepts differed among the nursing homes as follows: some training focused on person-centred care and related concepts, while other training focused on the management of aggression.

The attitude of employees should be flexible and adaptable, and employees should not expect a rigid notion of order. Employees should also be empathetic, engage in eye contact with the residents, and demonstrate that they do not feel superior to the residents. Both the representatives of the nursing homes and two representatives of the cost bearers reported that a better staff-to-resident ratio in dementia special care units is a prerequisite for implementing the concepts of special dementia care, which especially applies to situations occurring during the night, when staffing is extremely low.

#### Context—Organisation (design and environment)

The design of most dementia special care units supports segregation. The units have their own entrance, outdoor areas and kitchen. Nevertheless, the residents are allowed to leave the unit and walk to common areas in the nursing home. Some interviewees emphasised the necessity to have small groups to create a familiar atmosphere, while other interviewees thought wide spaces and areas were beneficiary because the residents had the need to move and walk around.

#### Context—Individual level (residents)

The residents of dementia special care units were described by the interviewees as people with dementia who exhibit severe responsive behaviour that overburden lay caregivers or caregivers in other nursing homes. Such behaviours include aggression, anxiety and withdrawal and are unstable and unpredictable in nature.

#### Dementia-specific interventions

We categorised the dementia-specific interventions described by the interview participants as follows:

- Activities
- Family and public involvement
- Behaviour management
- Environment

It became apparent from the descriptions of how these interventions are provided that they are not clearly separable but rather merge in daily life. For example, certain activities are designed to minimise stressors and the responsive behaviour.

In the nursing homes represented by the interview participants, *activities* were provided mainly by activity attendants. Activity attendants are not nurses, but are additional staff who are employed exclusively to spend time engaging the residents in activities. In Germany, their task spectrum is defined by a social insurance code (SGB XI § 43b, especially 53c) and encompasses activities expected to improve the physical and mental health of the residents and their quality of life. The *activities* focus on everyday tasks, such as cooking, gardening, reading or going to church. The *activities* are usually planned to occur at a certain time and are offered mostly as group activities or spontaneously. The interviewees described the *activities* as needing to be based on the preferences of the residents and noted that residents who fit well together are grouped together in *activities*.

The significance of *family involvement* was described very differently by the interviewees and seemed to depend on the target group of the dementia special care unit. In care units for residents with very severe cognitive impairments or responsive behaviour or have a rare forms of dementia, only a few relatives are present, whereas in other care units, there is a very strong relationship with relatives. The relationship between a family member and the nursing home extends even beyond the death of the family member. Active family work was described as involvement in activities, such as celebrating special events together. Relatives were considered a part of the community, actively contributing to social interaction and being involved in the care and support of their family members with dementia.

The interviewees commented that relatives of residents living in dementia special care units express much appreciation for the employees; this sentiment was described as stronger than that in other care units. Relatives value the work of carers in dementia special care units due to the strong emotional strain they have experienced and the freedom they are currently experiencing.

One participant reported that after a resident moves into a dementia special care unit, the situation must be accepted by the relatives, which includes explaining to the relatives the concept of the dementia special care unit. It is unusual for relatives not to correct the behaviour of the resident and encourage them to become independent, which also includes encouraging acceptance of the behaviour of other residents.

*Behaviour management* was described by the participants as including the assessment of responsive behaviour when a resident moves into the dementia special care unit and within case conferences when extraordinary situations arise. The interviewees did not provide further details regarding the management of responsive behaviour.

Using the *environment* as an intervention was described mostly as measures taken to personalise residents’ private rooms and common areas and adapt those areas to the residents’ needs. The adaptations consisted of the use of personal belongings and the design of common rooms. It became apparent that the adaptation of the *environment* had different targets as follows: some units attempted to avoid distraction triggered by the environment as much as possible, while other units created stimuli by decorating the *environment* with objects that the residents were familiar with from their past.

*Outcomes*. Enjoying life despite constraints related to taking psychotropic drugs was understood as quality of life and identified by the interviewees as an objective of dementia special care units. The use of neuroleptics was regarded as a constraint, and a goal for the promotion of the quality of life was a low level of neuroleptic use. Quality of life was also understood as being at peace with oneself and being balanced.

Another goal was to stabilise responsive behaviours to avoid extreme behaviours, such as aggression, while also accepting the behaviours of the residents. While certain behaviours cause serious problems in traditional care units and are repressed by staff or other residents, they are tolerated in dementia special care units.

Another aim was to activate the skills of the residents in a supportive way to promote independence and autonomy. One example was sustaining mobility, which was regarded as one of the most important prerequisites for autonomy.

### Description of the included studies

**Fig 2:**
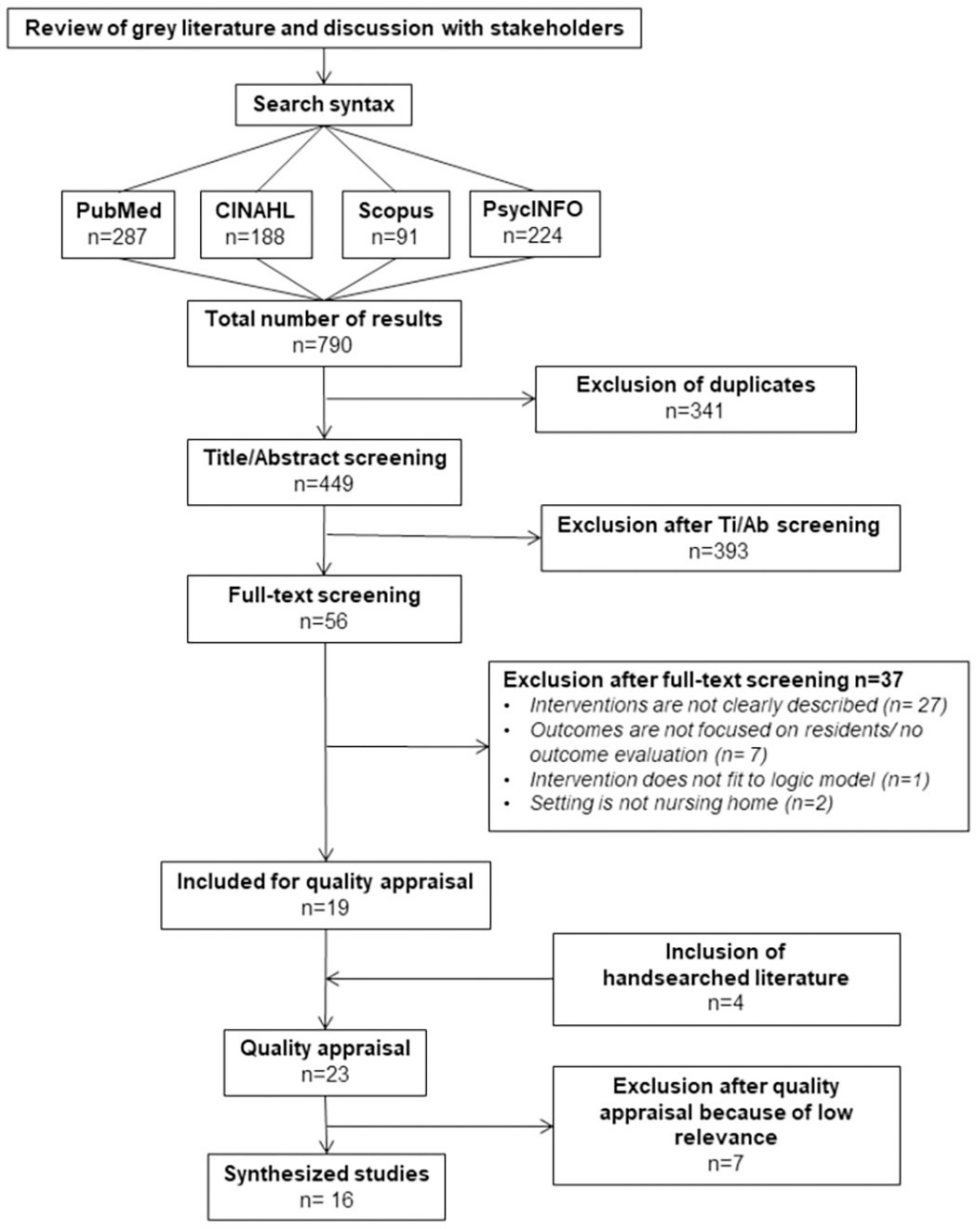
Flow chart of the interview and review process.

The systematic literature search identified n = 449 studies (without duplicates). After screening and the quality appraisal, we synthesised 16 articles from eight empirical studies as shown in Fig 2: Flow chart of the interview and review process. A list of the included studies is shown in Table 3: Characteristics of included studies, and the studies that were excluded after the full-text screening are listed in the supplementary material (S3 and S4 Tables). From each study, we developed CMO-configurations, which are shown in the supplementary material (S5 Table).

**Table 3:** Characteristics of the included studies.

Nr.	First author	Year	Country	Study design	Complex intervention	Intermediate/Process outcomes	Long-term outcomes
1.	Abbott, K.M. [34]	2017	USA	Observational pilot study	Activities Environment	Social integration	Quality of life
2.	Abbott, K.M. [35]	2017 a	USA	Observational pilot study	Activities Environment	Social integration	Quality of life
3.	Appelhof, B. [36]	2018	NL	Process evaluation	Behaviour management	Sample quality Intervention quality Implementation	
4.	Appelhof, B. [37].	2019	NL	Cluster RCT	Behaviour management	Psychotropic drug use	Behaviour
5.	De Boer, B. [38]	2017	NL	Longitudinal observational design with 3 comparison groups	Activities Environment (green care farms)	(Physical) activity involvement Social interaction	Quality of life
6.	De Boer, B. [39]	2017 a	NL	Cross-sectional observational design with 3 comparison groups	Activities Environment (green care farms)	Quality of care	Quality of life
7.	Helgesen, A.K. [40]	2010	Norway	Qualitative study using grounded theory	Activities	Residents’ participation	Autonomy
8.	Helgesen, A.K. [41]	2014	Norway	Qualitative study using grounded theory	Activities	Residents’ participation	Self-esteem and dignity
9.	Kok, J. [42]	2018	NL	Longitudinal observational design with 2 comparison groups	Activities Environment (small-scale living) Staff education		Quality of life Behaviour
10.	Smit, D. [43]	2012	NL	Cross-sectional study	Activities Environment (small-scale living)	Activity involvement	Quality of life
11.	Smit, D. [44]	2017	NL	Cross-sectional study	Activities	Activity involvement	Quality of life
12.	Verbeek, H. [45]	2010	NL	Longitudinal observational design with 2 comparison groups	Activities Environment (small-scale living)		Quality of life Behaviour Agitation
13.	Verbeek, H. [46]	2012	NL	Cross-sectional study (mixed methods)	Activities Environment (small-scale living)	Activity involvement Residents’ participation	Autonomy
14.	Verbeek, H. [47]	2014	NL	Longitudinal observational design with 2 comparison groups	Activities Environment (small-scale living)	Use of psychotropic drugs and physical restraints	Behaviour Agitation Depression Social engagement
15.	Zwijsen, S.A. [48]	2014	NL	Process evaluation	Behaviour management	Intervention fidelity and implementation	
16.	Zwijsen, S.A. [49]	2014 a	NL	Cluster RCT	Behaviour management	Use of psychotropic drugs	Behaviour

### Theory of action (realist informed logic model)

**Fig 3:**
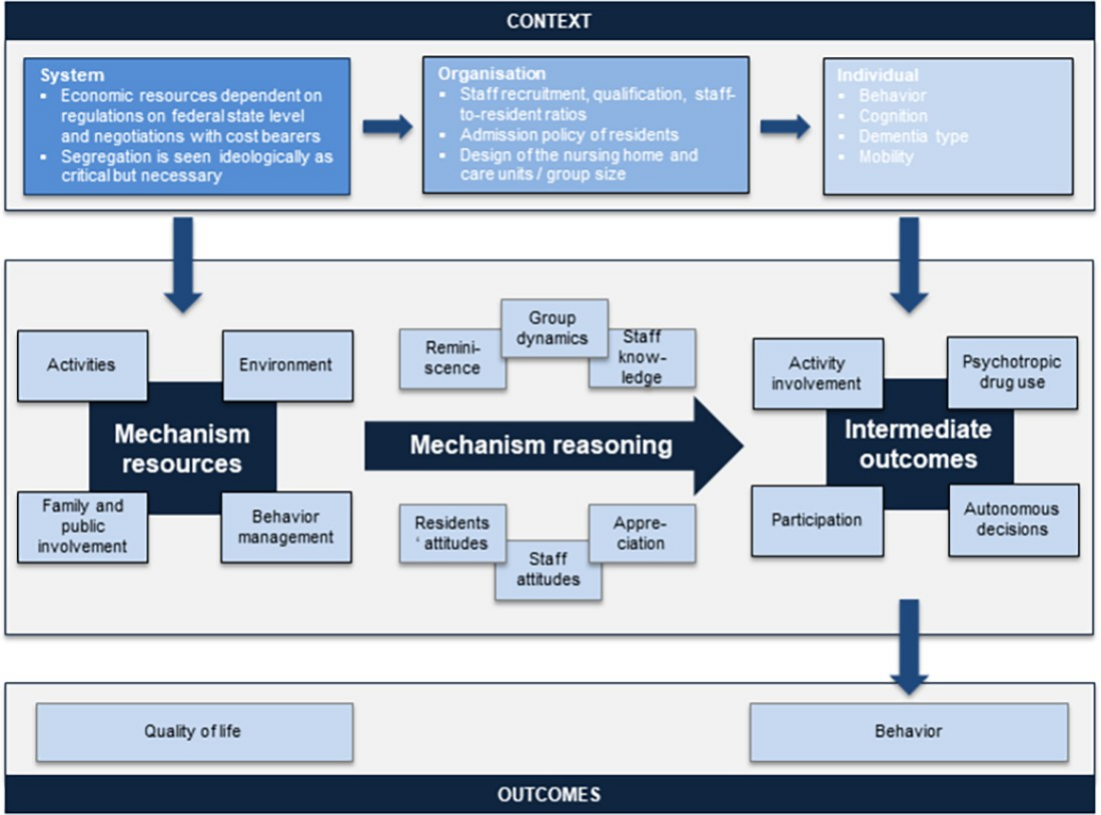
Theory of action (realist informed logic model).

The depicted theory of action is shown in Fig 3, which summarises the knowledge generated in the included empirical studies and the stakeholder interviews. Additionally, Fig 3 includes the anticipated mechanisms that we developed as a part of the theory of change.

### Theory of change (CMO configurations)

We present three CMO configurations that explain why a change in outcomes occurs when an intervention is implemented or received and which contextual factors of dementia special care units influence the underlying mechanisms of the interventions. We developed CMO configurations for environment adaptation, activity and behaviour management interventions.

The presented CMO configurations are a summary of the single CMO configurations that we developed from the included studies and the interviews (these CMO configurations are provided in the supplementary material, S5 Table). We were unable to extract CMO configurations for each intervention in the empirical studies and interviews. Information regarding family and public involvement in dementia-specific interventions was not found in the included empirical studies; therefore, the data were not rich enough to develop a CMO configuration.

1. **CMO configuration of the “environment” intervention**. *Context*. Regulations at the federal state/municipal and organisational level support the adaptation of the nursing home environment to the needs of residents with dementia. The existing buildings of nursing homes are or allow an adaptation of the architecture that is state of the art regarding dementia-sensitive buildings. The care units in the nursing home are designed and equipped in a way that facilitates residents’ activities by avoiding barriers, improves orientation and creates familiar places (e.g., a kitchen in the care unit). The context with respect to the physical environment is supportive.
  *Mechanism*. Residents are free of anxiety when moving independently in the care unit and recognise familiar features of the environment that remind them of activities they formerly performed in their life. The residents feel comfortable in the environment, associate positive feelings with the things they see and feel and recognise them as having personal value. The residents’ activities are triggered by the environment because the environment is related to their biography but does not cause an overreaction because of too much or negative stimulation.
  *Intermediate outcomes*. The environment encourages residents to become active on their own and interact with other residents or their family members and staff. The residents decide autonomously what to do and where to go.
  *Long-term outcomes*. Residents with dementia experience a positive change after they move in with respect to quality of life outcomes and responsive behaviour, suggesting that they show more positive affect, fewer verbal and physical behavioural symptoms of dementia and fewer symptoms of depression. Residents with dementia adapt successfully to their environment.
2. **CMO configuration of the “activity” intervention**. *Context*. Staff initiates and/or supervises spontaneously and/or regularly organised activities with the residents that are performed either individually or in group sessions according to the residents’ capabilities and resources. Staff qualitative and quantitative resources (knowledge and workload) are crucial for the provision of individual activities. The staff needs to be interested in and know the residents’ preferences to respect them; the staff must value what the residents do and say regardless of societal norms; and the staff must have the skills to motivate residents to be active. Employees who have been specifically selected to work at a dementia special care unit may be more likely to meet these requirements than those who were randomly assigned. For the provision and supervision of activities, a critical number of staff members is needed. The context with respect to the staff can be supportive or non-supportive.
  *Mechanism*. Staff members prioritise the resources, competencies and needs of individual residents and the entire resident group. According to the residents’ competencies, staff members can interpret the residents’ behaviour and expressions to communicate with the residents and offer activities that suit their needs. Staff members’ attitude influences their way of interacting with residents as either more paternalistic or more respectful. Staff members acknowledge who likes what and who fits well together in a group and organise activities accordingly. Special attention is given to residents who cannot communicate their preferences. Residents who prefer to engage in a certain activity and who are accepted and appreciated by the other residents in a group will feel comfortable performing group activities and will be more willing to participate; residents who do not feel accepted by the other group members or feel uncomfortable in the group will be more willing to engage in individual sessions if they like the provided activity.
  *Intermediate outcomes*. The residents decide whether they want to be active, what they want to do, when and with whom. The residents participate in group or individual activities based on their preferences.
  *Long-term outcomes*. The residents’ quality of life improves with respect to domains associated with activities and social relations. Over time, the residents show responsive behaviour less often.
3. **CMO configuration of the “behaviour management” intervention**. *Context*. The residents show unpredictable, severe responsive behaviour when they are admitted to the nursing home because this may be an admission criterion. Staff are trained to assess (un)obtrusive symptoms of severe responsive behaviour while identifying the reasons and reflecting upon alternatives to psychotropic drugs and can use various tools for a systematic assessment. If admission policies define severe responsive behaviours as a criterion, the residents may have to leave the unit when this behaviour becomes less severe. Staff fluctuation is low, and interdisciplinary cooperation is ensured.
  *Mechanism*. The staff differentiates the severity and types of responsive behaviours and the impact such behaviour has on the resident and others. The staff knows about the consequences of psychotropic drugs and appropriate alternatives (e.g., psychosocial interventions). The staff see value in their work in assessing behaviour and exchanging and reflecting upon interdisciplinary case conferences. The staff are able to make a decision regarding whether a behaviour needs to be treated and do not automatically attempt to reduce all behaviours that a resident displays.
  *Intermediate outcomes*. The behaviour of residents with dementia is assessed with regard to the severity and impact, and all staff members are aware of the assessment. For residents with severe responsive behaviour that needs to be treated, a care plan is available that contains alternative options to psychotropic drugs. The staff members weigh the potential harm of a psychotropic treatment against the harm the behaviour may cause to the resident and others. The staff may accept certain behaviours that might be suppressed in non-dementia special care units.
  *Long-term outcomes*. Residents who show unpredictable severe responsive behaviour when they move into the nursing home, show these behaviours less often over a longer period. Responsive behaviours that place the resident or others at risk of harm are prevented.

## Discussion

This study provides a comprehensive overview of the elements of dementia special care units for people with dementia and proposes how they influence each other. Comprehensiveness was assured by combining data from interviews and empirical studies that revealed different insights and perspectives. The interview data provided detailed information regarding contextual factors but were less informative regarding dementia-specific interventions and outcomes. Therefore, the information from the empirical studies was a very good supplement as the studies provided more details regarding how interventions are provided and outcomes are operationalised and measured. By combining the two data sources, we were able to abstract hidden mechanisms that we assume work in the background. We extracted the mechanisms mainly from the discussion part of the empirical studies, the results of process evaluations and the interview data. We show the mechanism in the realist informed logic model and the CMO configurations; both are parts of our initial programme theory.

In summary, the interventions and outcomes that were reported to be of relevance by the interviewees, except for the intervention to enhance family and public involvement, were also the focus of the included empirical studies. We could not identify any study that matched our criteria that evaluated interventions related to family and public involvement in dementia special care units. Similarly, the interviewees made very brief statements regarding their understanding of quality of life and how they assessed the success or failure of interventions with respect to this outcome. Regarding the behavioural outcomes, the interviewees were more specific and explained that their aim was to avoid unpredictable extreme behaviour that is harmful to others, whereas other forms of behaviour were accepted and not suppressed. The included empirical studies mostly aimed to prove a general decline in behavioural symptoms measured with multidimensional instruments that capture very different forms of such symptoms. We suspect that there may be a mismatch between the aims that clinicians pursue and the outcomes that empirical studies measure.

The realist informed logic model provides an overview that aims to inform the reader about context elements crucial for dementia special care units and the provision of dementia-specific interventions. To understand the relationships among these elements, a more detailed explanation is provided in the CMO configurations. Three CMO configurations explicate the reasoning of people who are actively involved in the following interventions that are typically applied for people with dementia in nursing homes: adaptation of the environment, provision of activities and behaviour management.

### Contextual elements that influence the intervention and outcomes

Below, we highlight four main contextual elements derived from both interviews and empirical studies that according to our initial programme theory, influence the provision of these dementia-specific interventions and their respective (intermediate) outcomes:

1. Resources and regulation: coherent and transparent policy
  The presence and absence of guiding policies, i.e., nationwide, state-wide, municipal, and organisation-wide, may determine the quality of care and outcome quality in dementia special care units. Because there is wide variation in nursing home and dementia care policies and regulations in Germany, the influence of these policies is not obvious and, in some aspects, indirect. Obtaining insight into these policies and regulations is not an easy task because in Germany, not all policies and regulations are publicly available. The transparency of regulations may also affect staff and consumer attitudes towards and expectations of dementia special care units; thus, this aspect also needs to be considered when dementia special care units are evaluated [50]. Studies have found that stringent quality regulations lead to better quality of care [51], and although the implementation of care standards and necessary monitoring is a matter of complaints (not only in Germany), they are linked to improvements in care processes and outcome quality [52]. The variation in dementia care policies that regulate dementia special care units is not a German phenomenon but also exists in other countries with a federal structure [50]. We assume that the (non)existence of policies for the regulation of dementia care is an influencing contextual factor because policies enable the accessibility of resources, influence the professional behaviour and attitudes of care staff and thereby indirectly influence residents’ observable and non-observable characteristics.
2. Segregation and admission policies: conflict of aims
  Segregation is a fundamental context issue because it has many implications. One aspect of segregation that was apparent in the interviews was striking, and we think that this aspect should be given special attention when the aim is to explain why context influences mechanisms. Segregation was not reported to cause much conflict with regard to the inclusion of people with dementia, but we noticed that it may cause a conflict of aims among nursing home directors and care practitioners. If segregation is regulated by admission policies, the residents have to leave the segregated care unit when they no longer fulfil the admission criteria, which is not compatible with the basic idea that nursing homes should provide a place that feels like home. Nursing home directors have to force a transfer if a resident or their relatives refuse to accept this and care practitioners have to put this into practice even if they do not support the underlying regulation. Otherwise, staff have to look for creative solutions to avoid transfers. We conclude that this fact should be given special attention when it is required to explain why context influences mechanisms, because admission policies have a strong impact on the attitude of the staff and their corresponding reaction.
3. Staff: purposeful recruitment and education of staff
  How staff members are recruited and educated in dementia special care units may be an influencing contextual factor. Purposeful selection and intensified education may impact the professional attitude and identity of nurses. Studies have shown that staff members in dementia special care units experience less work stress and less exposure to physical assault than staff members in other care units, although the contrary may be expected [53]. We assume that staff members working in a dementia special care unit may be “protected” by a stronger commitment to their workplace, a higher level of education-based skills and more positive feedback from residents and relatives and, thus, show better work-related outcomes. We did not find any studies supporting this assumption and, therefore, recommend considering it in future studies.
4. A good fit between the environment and the individuals
  The reciprocal ability to adapt plays an important role as follows: residents’ ability to adapt to the new environment and the environment’s ability to adapt to the residents. The environment includes both physical and social environments. It is apparent that different residents need different environments, and the challenge is to satisfy these different needs. Staff members need the competence to ensure a good fit between individual preferences and collective interests, and the residents are required to respect other residents’ preferences even if they do not align with their own. This phenomenon is particularly apparent when activities are provided in a group. The mediating effects of relatedness, self-determined motivation and adaptation were studied by Altintas et al. [54], who showed that feeling connected and secure in relationships with others and integrated as an individual in a group contributed to participation in activities, motivation and finally adaptation to the nursing home. We, therefore, assume that a good fit between the residents living in a dementia special care unit is a contextual factor that influences dementia-specific interventions, especially activities.

#### Strengths and limitations

The realist review and stakeholder interviews, which are the basis of the initial programme theory, are not without limitations that may have affected the results. The realist review is not a comprehensive literature review; therefore, studies that were relevant to the topics of the review may not have been included. Many intervention studies did not report whether they were conducted in a dementia special care unit, which is why we excluded them. Not having reported the setting does not automatically mean that the studies were not performed in a dementia special care unit. The exclusion of studies because of low relevance for theory building may also be a limitation. The assessment of relevance was performed by two researchers, and studies were excluded only if the researchers agreed, but nonetheless, this process reduced information for theory building.

The stakeholder interviews were initially not performed to develop a realist initial programme theory, but the idea emerged when the interviews were analysed. Therefore, the interviews were not performed following realist interviewing principles, which may explain why they were not as rich in content regarding the connection among context, mechanism and outcomes.

Not including nurses and other care providers may be considered a limitation because these individuals may have given richer information regarding the provision of dementia-specific interventions. However, we think that asking for this type of implicit knowledge is a challenge in general.

The strength of this initial program theory is the combination of empirical literature with findings from empirical interviews.

### Conclusion and future implications for research

Because the presented programme theory is an initial one, we formed no conclusions regarding its impact on the provision of care, but we outline how future research should be designed to further develop the initial programme theory, test the assumed CMO configurations and develop new ones and finally achieve a higher level of validity and theoretical abstraction.

Future studies should explore whether the formulated CMO configurations hold in different contexts or whether they need to be revised. Of particular interest in this respect are the contextual factors “coherence and transparency of policies”, “purposeful recruitment and education of staff” and “a good fit between the environment and the individuals”. This exploration should involve formulating testable hypotheses derived from the CMO configurations and testing them using appropriate empirical methods. We consider the involvement of care practitioners (nurses and activity attendants), relatives and residents and the application of different methods to assess the hypotheses absolutely necessary for this theory development. For other researcher, we recommend to describe clearly their setting of research with respect to the characteristics of a dementia special care unit. This would enhance the understanding of CMO configurations in dementia special care units.

By conducting a multistage process of empirical research, theory building and testing, we seek to develop a programme theory that can be used by researchers and practitioners or providers of care. Researchers in the field of intervention and implementation research in the context of dementia special care units can base their studies on the theory and model the assumed mechanism of action. For practitioners and care providers, programme theory may be helpful in developing organisational policies and guidelines or as a basis for internal quality management.

## Supporting information

S1 Checklist RAMESES checklist. (DOCX)

S1 Table Categorisation scheme of the interviews. (DOCX)

S2 Table Search syntax for the systematic literature search. (DOCX)

S3 Table List of excluded studies after fulltext screening. (DOCX)

S4 Table List of excluded studies after quality appraisal. (DOCX)

S5 Table CMO-configurations developed from included studies and interviews. (DOCX)

S1 File Quality appraisal. (DOCX)
