# Supplementary material for: Context, mechanisms and outcomes of dementia special care units: An initial programme theory based on realist methodology
Source: PLoS One. 2021 Nov 16;16(11):e0259496. doi: 10.1371/journal.pone.0259496 (PMC8594822; doi:10.1371/journal.pone.0259496)
Supplement: S1 Checklist — (DOCX) [file pone.0259496.s001.docx]

|  |  | Manuscript reference |
| --- | --- | --- |
| **TITLE** |  |  |
| 1 | In the title, identify the document as a realist synthesis or review | Because the manuscript combines a realist review with interviews, we refer in the title to the realist methodology. |
| **abstract** |  |  |
| 2 | While acknowledging publication requirements and house style, abstracts should ideally contain brief  details of: the study’s background, review question or objectives; search strategy; methods of selection,  appraisal, analysis and synthesis of sources; main results; and implications for practice. | Items are acknowledged in the abstract, except “implications for the practice”. Because the manuscript reports an initial program theory, we do not recommend implications for the practice. |
| **introduction** |  |  |
| 3 Rationale for review | Explain why the review is needed and what it is likely to contribute to existing understanding of the  topic area. | Page 3 line 40 to page 5 line100 |
| 4 Objectives and focus of review | State the objective(s) of the review and/or the review question(s). Define and provide a rationale for the focus of the review. | Page 5 line 97-100 and page 7 line 154 – page 8 line 178 |
| **Methods** |  |  |
| 5 Changes in the review process | Any changes made to the review process that was initially planned should be briefly described and  justified. | Not applicable. |
| 6 Rationale for using realist synthesis | Explain why realist synthesis was considered the most appropriate method to use. | Page 8 line 181 - 183 |
| 7 Scoping the literature | Describe and justify the initial process of exploratory scoping of the literature. | Page 8 line 183 - 186 |
| 8 Searching processes | While considering specific requirements of the journal or other publication outlet, state and provide a rationale for how the iterative searching was done. Provide details on all the sources accessed for information in the review. Where searching in electronic databases has taken place, the details should include, for example, name of database, search terms, dates of coverage and date last searched. If individuals familiar with the relevant literature and/or topic area were contacted, indicate how they were identified and selected. | Page 10 line 232-254  Table 1, Figure 2, Supporting material S1, S3, S4  Page 8 line 186 - 187 |
| 9 Selection and appraisal of  documents | Explain how judgements were made about including and excluding data from documents, and justify  these. | Table 1  Supporting material S3, S4 |
| 10 Data extraction | Describe and explain which data or information were extracted from the included documents and justify this selection. | Because the realist review is combined with interviews and part of the development of an initial program theory we describe this process on page 8 line 181- page 10 line 220 including figure 1. |
| 11 Analysis and synthesis processes | Describe the analysis and synthesis processes in detail. This section should include information on the constructs analyzed and describe the analytic process. | Page 8 line 181- page 10 line 220 including figure 1. |
| **RESULTS** |  |  |
| 12 Document flow diagram | Provide details on the number of documents assessed for eligibility and included in the review with reasons for exclusion at each stage as well as an indication of their source of origin (for example, from searching databases, reference lists and so on). You may consider using the example templates (which are likely to need modification to suit the data) that are provided. | Figure 2 |
| 13 Document characteristics | Provide information on the characteristics of the documents included in the review. | Table 3 |
| 14 Main findings | Present the key findings with a specific focus on theory building and testing. | The key findings are presented as context-mechanism-outcome configurations on page 21-23 |
| **DISCUSSION** |  |  |
| 15 Summary of findings | Summarize the main findings, taking into account the review’s objective(s), research question(s), focus and intended audience(s). | Page 25 line 479 – page 26 line 518 |
| 16 Strengths, limitations and future  research directions | Discuss both the strengths of the review and its limitations. These should include (but need not be restricted to) (a) consideration of all the steps in the review process and (b) comment on the overall strength of evidence supporting the explanatory insights which emerged. The limitations identified may point to areas where further work is needed. | Page 26 line 519 – page 27 line 534 |
| 17 Comparison with existing literature | Where applicable, compare and contrast the review’s findings with the existing literature (for example, other reviews) on the same topic. | Page 25 line 479 – page 26 line 518 |
| 18 Conclusion and recommendations | List the main implications of the findings and place these in the context of other relevant literature. If appropriate, offer recommendations for policy and practice. | Page 27 line 535 - 551 |
| 19 Funding | Provide details of funding source (if any) for the review, the role played by the funder (if any) and any conflicts of interests of the reviewers. | The study did not receive external funding.  Page 28 line 554-555 |

Table: List of items to be included when reporting a realist synthesis

Reference to the checklist:

<https://bmcmedicine.biomedcentral.com/articles/10.1186/1741-7015-11-21>
