# Supplementary material for: Context, mechanisms and outcomes of dementia special care units: An initial programme theory based on realist methodology
Source: PLoS One. 2021 Nov 16;16(11):e0259496. doi: 10.1371/journal.pone.0259496 (PMC8594822; doi:10.1371/journal.pone.0259496)
Supplement: S1 Table — (DOCX) [file pone.0259496.s002.docx]

| **Category** | **Subcategory** |
| --- | --- |
| Characteristics of the segregated care unit | *Organisation* |
|  | *Architecture and environment* |
|  | *Interventions for the integration in the community* |
|  | *Drug management* |
|  | *Social care & activities* |
|  | *Psychosocial care* |
|  | *Case conferences* |
|  | *Nursing and medical diagnostics* |
|  | *Therapeutics* |
|  | *Collaboration with physicians* |
|  | *Discharge management* |
|  | *Work with relatives* |
|  | *Staff qualification* |
|  | *Staff resources* |
|  | *Measures to improve security* |
|  |  |
| Target group | *Dementia type* |
|  | *Behaviour* |
|  | *Mobility* |
|  | *Korsakow* |
|  | *Age* |
|  | *Legal court order* |
|  | *Palliative phase* |
|  | *Composition of the group* |
|  |  |
| Goals of the segregated care unit | *Feel at home* |
|  | *Avoid suffering* |
|  | *Avoid danger* |
|  | *To be who you are* |
|  | *Reduction of psychoactive drugs* |
|  | *Freedom* |
|  | *Recover resources* |
|  | *Quality of life* |
|  | *Reduce behaviour abnormalities* |
|  |  |
| Reason for the implementation of the segregated care unit | *Demand of places* |
|  | *Specialists on the market* |
|  | *Differentiation from others* |
|  | *Avoidance of re-admissions* |
|  | *Improve care* |
|  | *Valuing work* |
|  | *Financial reasons* |
|  | *Not enough staff* |
| External quality management | *Medical Review Board of the Statutory Health Insurance Funds* |
|  | *External audits by inspectors of health authorities* |
|  | *Hygiene control* |
|  |  |
| Economics | *Financing* |
|  | *Costs* |
|  | *Financial benefit* |
|  |  |
| Regulations | *Implementation of regulations* |
|  | *Role of cost bearers* |
|  | *Transparency of regulations* |
|  | *Usefulness* |
|  |  |
| Experienced problems | *Staff shortage* |
|  | *Care provision in hospitals* |
|  | *Administration* |
|  | *Cost absorption* |
|  | *Contract negotiations* |
|  | *Compliance with requirements* |
|  | *Recognition of the special care concept* |
|  | *Attitude of staff* |
|  | *Qualification of staff* |
|  | *Concept specification* |
|  |  |

Table S 1: Categorisation scheme of the interviews
