# Supplementary material for: Context, mechanisms and outcomes of dementia special care units: An initial programme theory based on realist methodology
Source: PLoS One. 2021 Nov 16;16(11):e0259496. doi: 10.1371/journal.pone.0259496 (PMC8594822; doi:10.1371/journal.pone.0259496)
Supplement: S2 Table — (DOCX) [file pone.0259496.s003.docx]

|  |  |  |
| --- | --- | --- |
| **Database: PubMed** | | |
| **thematic block** | **no.** | **search term** |
| **scu** | #1 | special care unit* [Title/Abstract] |
|  | #2 | scu [Title/Abstract] |
|  | #3 | special care dementia unit* [Title/Abstract] |
|  | #4 | special care ward* [Title/Abstract] |
|  | #5 | special care area* [Title/Abstract] |
|  | #6 | special care facilit* [Title/Abstract] |
|  | #7 | small-scale facilit* [Title/Abstract] |
|  | #8 | small-scale living [Title/Abstract] |
|  | #9 | small-scale care [Title/Abstract] |
|  | #10 | small-scale unit [Title/Abstract] |
|  | #11 | specific care unit* [Title/Abstract] |
|  | #12 | "specialised living unit*" [Title/Abstract] |
|  | #13 | "specialized living unit*" [Title/Abstract] |
|  | #14 | homelike care [Title/Abstract] |
|  | #15 | "homelike living" [Title/Abstract] |
|  | #16 | homelike facilit* [Title/Abstract] |
|  | **#20** | **#1 OR #2 OR #3 OR #4 OR #5 OR #6 OR #7 OR #8 OR #9 OR #10 OR #11 OR #12 OR #13 OR #14 OR #15 OR #16** |
|  | | |
| **dementia** | #18 | dementia [Title/Abstract] |
|  | #19 | dement* [Title/Abstract] |
|  | #20 | dementia [MeSH Terms] |
|  | #21 | Alzheimer* [Title/Abstract] |
|  | **#22** | **#18 OR #19 OR #20 OR #21** |
|  | | |
| **ltc** | #23 | long term care [Title/Abstract] |
|  | #24 | longterm care [Title/Abstract] |
|  | #25 | ltc [Title/Abstract] |
|  | #26 | care home* [Title/Abstract] |
|  | #27 | care facility [Title/Abstract] |
|  | #28 | care facilities [Title/Abstract] |
|  | #29 | nursing facilit* [Title/Abstract] |
|  | #30 | residential homes [Title/Abstract] |
|  | #31 | residential home [Title/Abstract] |
|  | #32 | residential care[Title/Abstract] |
|  | #33 | "residential facility" [Title/Abstract] |
|  | #34 | "residential facilities" [Title/Abstract] |
|  | #35 | assisted living facilit* [Title/Abstract] |
|  | #36 | residential aged care [Title/Abstract] |
|  | #37 | home for the elderly [Title/Abstract] |
|  | #38 | homes for the elderly [Title/Abstract] |
|  | #39 | home for the aged[Title/Abstract] |
|  | #40 | homes for the aged[Title/Abstract] |
|  | #41 | group home*[Title/Abstract] |
|  | #42 | nursing home*[Title/Abstract] |
|  | #43 | inpatient [Title/Abstract] |
|  | #44 | in-patient [Title/Abstract] |
|  | #45 | residential facilities [MeSH Terms] |
|  | **#46** | **#23 OR #24 OR #25 OR #26 OR … OR #45** |
|  | | |
|  | **#47** | **#22 AND #46** |
|  | **#48** | **#22 AND #46 AND #17** |

| **Database: CINAHL** | | |
| --- | --- | --- |
| **thematic block** | **no.** | **search term** |
| **scu** | #1 | TI "special care unit*" OR AB "special care unit*" |
|  | #2 | TI scu OR AB SCU |
|  | #3 | TI "special care dementia unit*" OR AB "special care dementia unit*" |
|  | #4 | TI "special care ward*" OR AB "special care ward*" |
|  | #5 | TI "special care area*" OR AB "special care area*" |
|  | #6 | TI "special care facilit*" OR AB "special care facilit*" |
|  | #7 | TI "small scale* facilit*" OR AB "small scale* facilit*" |
|  | #8 | TI "small scale* living" OR AB "small scale* living" |
|  | #9 | TI "small scale* care" OR AB "small scale* care" |
|  | #10 | TI "small scale* unit*" OR AB "small scale* unit*" |
|  | #11 | TI "specific care unit*" OR AB "specific care unit*" |
|  | #12 | TI "specialised unit*" OR AB "specialised unit*" |
|  | #13 | TI "specialized unit*" OR AB "specialized unit*" |
|  | #14 | TI "homelike care" OR AB "homelike care" |
|  | #15 | TI "homelike living" OR AB "homelike living" |
|  | #16 | TI "homelike facilit*" OR AB "homelike facilit*" |
|  | #17 | **#1 OR #2 OR #3 OR #4 OR #5 OR #6 OR #7 OR #8 OR #9 OR #10 OR #11 OR #12 OR #13 OR #14 OR #15 OR #16 OR #17 OR #18 OR #19 OR #20** |
|  | | |
| **dementia** | #18 | TI dementia OR AB dementia |
|  | #19 | TI dement* OR AB dement* |
|  | #20 | MM dementia |
|  | #21 | TI alzheimer* OR AB alzheimer* |
|  | #22 | **#22 OR #23 OR #24 OR #25** |
|  | | |
| **ltc** | #23 | TI "long term care" OR AB "long term care" |
|  | #24 | TI "longterm care" OR AB "longterm care" |
|  | #25 | TI ltc OR AB ltc |
|  | #26 | TI "care home*" OR AB "care home*" |
|  | #27 | TI "care facilit*" OR AB " care facilit*" |
|  | #28 | TI "nursing facilit*" OR AB "nursing facilit*" |
|  | #29 | TI "assisted living facilit*" OR AB "assisted living facilit*" |
|  | #30 | TI "residential facilit*" OR AB "residential facilit*" |
|  | #31 | TI "residential care" OR AB "residential care" |
|  | #32 | TI "residential home*" OR AB "residential home*" |
|  | #33 | TI "residential aged care" OR AB "residential aged care" |
|  | #34 | TI "home* for the elderly" OR AB "home* for the elderly" |
|  | #35 | TI "home* for the aged" OR AB "home* for the aged" |
|  | #36 | TI "group home*" OR AB "group home*" |
|  | #37 | TI "nursing home*" OR AB "nursing home*" |
|  | #38 | TI inpatient OR AB inpatient |
|  | #39 | TI in-patient OR AB in-patient |
|  | #40 | MM residential facilities |
|  | #41 | **#27 OR #28 OR #29 OR #30 OR … OR #43** |
|  | | |
|  | **#42** | **#22 AND #41** |
|  | **#43** | **#22 AND #41 AND #17** |

| **Database: PsycINFO** | | |
| --- | --- | --- |
| **thematic block** | **no.** | **search term** |
| **scu** | #1 | TI special care unit OR AB special care unit |
|  | #2 | TI scu OR AB SCU |
|  | #3 | TI special care dementia unit OR AB special care dementia unit |
|  | #4 | TI special care ward OR AB special care ward |
|  | #5 | TI special care area OR AB special care area |
|  | #6 | TI special care facility OR AB special care facility |
|  | #7 | TI small-scale facility OR AB small-scale facility |
|  | #8 | TI small-scale living OR AB small-scale living |
|  | #9 | TI small-scale care OR AB small-scale care |
|  | #10 | TI small-scale unit OR AB small-scale unit |
|  | #11 | TI specific care unit OR AB specific care unit |
|  | #12 | TI specialised unit OR AB specialised unit |
|  | #13 | TI specialized unit OR AB specialized unit |
|  | #14 | TI homelike care OR AB homelike care |
|  | #15 | TI homelike living OR AB homelike living |
|  | #16 | TI homelike facility OR AB homelike facility |
|  | **#17** | **#1 OR #2 OR #3 OR #4 OR #5 OR #6 OR #7 OR #8 OR #9 OR #10 OR #11 OR #12 OR #13 OR #14 OR #15 OR #16** |
|  | | |
| **dementia** | #18 | TI dement* OR AB dement* |
|  | #19 | MA dementia |
|  | #20 | TI alzheimer OR AB alzheimer |
|  | **#21** | **#18 OR #19 OR #20** |
|  | | |
| **ltc** | #22 | TI long term care OR AB long term care |
|  | #23 | TI longterm care OR AB longterm care |
|  | #24 | TI ltc OR AB ltc |
|  | #25 | TI care home OR AB care home |
|  | #26 | TI care facility OR AB care facility |
|  | #27 | TI nursing facility OR AB nursing facility |
|  | #28 | TI assisted living facility OR AB assisted living facility |
|  | #29 | TI residential facility OR AB residential facility |
|  | #30 | TI residential care OR AB residential care |
|  | #31 | TI residential home OR AB residential home |
|  | #32 | TI residential aged care OR AB residential aged care |
|  | #33 | TI "home for the elderly" OR AB "home for the elderly" |
|  | #34 | TI "home for the aged" OR AB "home for the aged" |
|  | #35 | TI group home OR AB group home |
|  | #36 | TI nursing home OR AB nursing home |
|  | #37 | TI inpatient OR AB inpatient |
|  | #38 | TI in-patient OR AB in-patient |
|  | #39 | MA residential facilities |
|  | **#40** | **#22 OR #23 OR #24 OR #25 OR … OR #39** |
|  | | |
|  | **#41** | **#21 AND #40** |
|  | **#42** | **#21 AND #40 AND 17** |

| **Database: Scopus** | | |
| --- | --- | --- |
| **thematic block** | **no.** | **search term** |
| **scu** | #1 | ( TITLE ( "special care unit" )  OR  ABS ( "special care unit" ) ) |
|  | #2 | ( TITLE ( scu )  OR  ABS ( scu ) ) |
|  | #3 | ( TITLE ( "special care dementia unit" )  OR  ABS ( "special care dementia unit" ) ) |
|  | #4 | ( TITLE ( "special care ward" )  OR  ABS ( "special care ward" ) ) |
|  | #5 | ( TITLE ( "special care area" )  OR  ABS ( "special care area" ) ) |
|  | #6 | ( TITLE ( "special care facility" )  OR  ABS ( "special care facility" ) ) |
|  | #7 | ( TITLE ( "small scale* facility" )  OR  ABS ( "small scale* facility" ) ) |
|  | #8 | ( TITLE ( "small scale* living" )  OR  ABS ( "small scale* living" ) ) |
|  | #9 | ( TITLE ( "small scale* care" )  OR  ABS ( "small scale* care" ) ) |
|  | #10 | ( TITLE ( "small scale* unit" )  OR  ABS ( "small scale* unit" ) ) |
|  | #11 | ( TITLE ( "specific care unit" )  OR  ABS ( "specific care unit" ) ) |
|  | #12 | ( TITLE ( "specialised living unit" )  OR  ABS ( "specialised living unit" ) ) |
|  | #13 | ( TITLE ( "specialized living unit" )  OR  ABS ( "specialized living unit" ) ) |
|  | #14 | ( TITLE ( "homelike care" )  OR  ABS ( "homelike care" ) ) |
|  | #15 | ( TITLE ( "homelike living" )  OR  ABS ( "homelike living" ) ) |
|  | #16 | ( TITLE ( "homelike facility" )  OR  ABS ( "homelike facility" ) ) |
|  | **#17** | **#1 OR #2 OR #3 OR #4 OR #5 OR #6 OR #7 OR #8 OR #9 OR #10 OR #11 OR #12 OR #13 OR #14 OR #15 OR #16** |
|  | | |
| **dementia** | #18 | ( TITLE ( "dement*" )  OR  ABS ( "dement*" ) ) |
|  | #19 | ( TITLE ( "alzheimer*" )  OR  ABS ( "alzheimer*" ) ) |
|  | **#20** | **#18 OR #19** |
|  | | |
| **ltc** | #21 | ( TITLE ( "long term care" )  OR  ABS ( "long term care" ) ) |
|  | #22 | ( TITLE ( "longterm care" )  OR  ABS ( "longterm care" ) ) |
|  | #23 | ( TITLE ( ltc )  OR  ABS ( ltc ) ) |
|  | #24 | ( TITLE ( "care home" )  OR  ABS ( "care home" ) ) |
|  | #25 | ( TITLE ( "care facility" )  OR  ABS ( "care facility" ) ) |
|  | #26 | ( TITLE ( "nursing facility" )  OR  ABS ( "nursing facility" ) ) |
|  | #27 | ( TITLE ( "assisted living facility" )  OR  ABS ( "assisted living facility" ) ) |
|  | #28 | ( TITLE ( "residential facility" )  OR  ABS ( "residential facility" ) ) |
|  | #29 | ( TITLE ( "residential care" )  OR  ABS ( "residential care" ) ) |
|  | #30 | ( TITLE ( "residential home" )  OR  ABS ( "residential home" ) ) |
|  | #31 | ( TITLE ( "residential aged care" )  OR  ABS ( "residential aged care" ) ) |
|  | #32 | ( TITLE ( "home for the elderly" )  OR  ABS ( "home for the elderly" ) ) |
|  | #33 | ( TITLE ( "home for the aged" )  OR  ABS ( "home for the aged" ) ) |
|  | #34 | ( TITLE ( "group home" )  OR  ABS ( "group home" ) ) |
|  | #35 | ( TITLE ( "nursing home" )  OR  ABS ( "nursing home" ) ) |
|  | #36 | ( TITLE ( "inpatient" )  OR  ABS ( "inpatient" ) ) |
|  | #37 | ( TITLE ( "in-patient" )  OR  ABS ( "in-patient" ) ) |
|  | **#38** | **#21 OR #23 OR #24 OR #25 OR … OR #37** |
|  | | |
|  | **#41** | **#20 AND #38** |
|  | **#42** | **#20 AND #38 AND #17** |
