# Supplementary material for: Context, mechanisms and outcomes of dementia special care units: An initial programme theory based on realist methodology
Source: PLoS One. 2021 Nov 16;16(11):e0259496. doi: 10.1371/journal.pone.0259496 (PMC8594822; doi:10.1371/journal.pone.0259496)
Supplement: S3 Table — (DOCX) [file pone.0259496.s004.docx]

|  | References excluded after full text screening | Reason |
| --- | --- | --- |
| 1 | Abbott KM, Bettger JP, Hampton KN, et al. The feasibility of measuring social networks among older adults in assisted living and dementia special care units. Dementia: The International Journal of Social Research and Practice. 2015;14(2):199-219. | 2 |
| 2 | Abrahamson K, Clark D, Perkins A, et al. Does cognitive impairment influence quality of life among nursing home residents? The Gerontologist. 2012;52(5):632-640. | 1 |
| 3 | Abrahamson K, Lewis T, Perkins A, et al. The influence of cognitive impairment, special care unit placement, and nursing facility characteristics on resident quality of life. Journal of aging and health. 2013;25(4):574-588. | 1 |
| 4 | Adams J, Verbeek H, Zwakhalen SMG. The impact of organizational innovations in nursing homes on staff perceptions: A secondary data analysis. Journal of Nursing Scholarship. 2017;49(1):54-62. | 2 |
| 5 | Anderson K, Bird M, Blair A, et al. Development and effectiveness of an integrated inpatient and community service for challenging behaviour in late life: From confused and disturbed elderly to transitional behavioural assessment and intervention service. Dementia: The International Journal of Social Research and Practice. 2016;15(6):1340-1357. | 4 |
| 6 | Arens OB, Fierz K, Zúñiga F. Elder abuse in nursing homes: Do special care units make a difference? A secondary data analysis of the Swiss Nursing Homes Human Resources Project. Gerontology. 2017;63(2):169-179. | 1 |
| 7 | Aukner C, Eide HD, Iversen PO. Nutritional status among older residents with dementia in open versus special care units in municipal nursing homes: an observational study. BMC geriatrics. 2013;13:26. | 1 |
| 8 | Bjork S, Juthberg C, Lindkvist M, et al. Exploring the prevalence and variance of cognitive impairment, pain, neuropsychiatric symptoms and ADL dependency among persons living in nursing homes; a cross-sectional study. BMC geriatrics. 2016;16:154. | 1 |
| 9 | Brandenburg H. Lebensqualität von menschen mit schwerer demenz in pflegeoasen: Empirische ergebnisse und methodische implikationen. = Quality of life of people with severe dementia in nursing oases: Empirical results and methodological implications. Zeitschrift fur Gerontologie und Geriatrie. 2013;46(5):417-424. | 1 |
| 10 | Brandenburg H, Adam-Paffrath R, Guther H. [Aspects of quality of life of residents of a nursing oasis from the perspectives of relatives and nurses- qualitative results of an evaluation study]. Pflege. 2014;27(2):69-80. | 1 |
| 11 | Cadigan RO, Grabowski DC, Givens JL, et al. The quality of advanced dementia care in the nursing home: The role of special care units. Medical care. 2012;50(10):856-862. | 1 |
| 12 | Castle NG. Special care units and their influence on nursing home occupancy characteristics. Health care management review. 2008;33(1):79-91. | 1 |
| 13 | Crespo M, Hornillos C, Gómez MM. Dementia special care units: A comparison with standard units regarding residents' profile and care features. International psychogeriatrics. 2013;25(12):2023-2031. | 1 |
| 14 | de Rooij AH, Luijkx KG, Declercq AG, et al. Quality of life of residents with dementia in long-term care settings in the Netherlands and Belgium: design of a longitudinal comparative study in traditional nursing homes and small-scale living facilities. BMC geriatrics. 2011;11:20. | 2 |
| 15 | de Rooij AHPM, Luijkx KG, Declercq AG, et al. Professional Caregivers’ Mental Health Problems and Burnout in Small-Scale and Traditional Long Term Care Settings for Elderly People With Dementia in The Netherlands and Belgium. Journal of the American Medical Directors Association. 2012;13(5):486.e487-486.e411. | 2 |
| 16 | de Rooij AHPM, Luijkx KG, Schaafsma J, et al. Quality of life of residents with dementia in traditional versus small-scale long-term care settings: A quasi-experimental study. International journal of nursing studies. 2012;49(8):931-940. | 1 |
| 17 | Deufert D, Mantovan F, Huber S, et al. 'Living with instruction': Evaluation of a special care unit for people with dementia in South Tyrol. Pflegewissenschaft. 2013;15(3):145-150. | 1 |
| 18 | Edwards RB. Alzheimer's special care units: Dementia and Alzheimer's disease in residential care and assisted living facilities. US, ProQuest Information & Learning; 2017. | 1 |
| 19 | Fawcett EJ. Are Alzheimer's special care units really special? Effects of residential status on family members' perspectives on high quality care for their loved-ones in long-term care. US, ProQuest Information & Learning; 2013. | 1 |
| 20 | Fischer T, Wolf-Ostermann K. Die Berliner studie zu versorgungsstrukturen und versorgungsergebnissen von wohngemeinschaften für menschen mit demenz (DeWeGE). = The Berlin study on structures and outcomes of «Wohngemeinschaften» for people with dementia (DeWeGE), a small-scale living arrangement. Zeitschrift für Gerontopsychologie und -psychiatrie. 2008;21(3):179-183. | 4 |
| 21 | Gruneir A. Dementia special care units: Another look at their impact and role in the nursing home market. US, ProQuest Information & Learning; 2008. | 1 |
| 22 | Gruneir A, Lapane KL, Miller SC, et al. Does the presence of a dementia special care unit improve nursing home quality? Journal of aging and health. 2008;20(7):837-854. | 1 |
| 23 | Gruneir A, Lapane KL, Miller SC, et al. Is dementia special care really special? A new look at an old question. Journal of the American Geriatrics Society. 2008;56(2):199-205. | 1 |
| 24 | Kirkevold O, Eek A, Engedal K. Development of residential care services facilitated for persons with dementia in Norway. Aging clinical and experimental research. 2012;24(1):1-5. | 1 |
| 25 | Kok JS, Berg IJ, Scherder EJ. Special care units and traditional care in dementia: relationship with behavior, cognition, functional status and quality of life - a review. Dementia and geriatric cognitive disorders extra. 2013;3(1):360-375. | 1 |
| 26 | Lai CKY, Yeung JHM, Mok V, et al. Special care units for dementia individuals with behavioural problems. Cochrane Database of Systematic Reviews. 2009(4). | 1 |
| 27 | Luo H, Fang X, Liao Y, et al. Associations of special care units and outcomes of residents with dementia: 2004 National Nursing Home Survey. The Gerontologist. 2010;50(4):509-518. | 1 |
| 28 | McCarty CE. The impact of Hospice and Dementia Special Care Units on end-of-life care for individuals with dementia. US, ProQuest Information & Learning; 2012. | 3 |
| 29 | Nobili A, Piana I, Balossi L, et al. Alzheimer special care units compared with traditional nursing home for dementia care: Are there differences at admission and in clinical outcomes? Alzheimer disease and associated disorders. 2008;22(4):352-361. | 1 |
| 30 | Palm R, Bartholomeyczik S, Roes M, et al. Structural characteristics of specialised living units for people with dementia: a cross-sectional study in German nursing homes. International journal of mental health systems. 2014;8(1):39. | 1 |
| 31 | Park-Lee E, Sengupta M, Harris-Kojetin LD. Dementia special care units in residential care communities: United States, 2010. NCHS data brief. 2013(134):1-8. | 1 |
| 32 | Verbeek H, van Rossum E, Zwakhalen SM, et al. Small, homelike care environments for older people with dementia: a literature review. International psychogeriatrics. 2009;21(2):252-264. | 2 |
| 33 | Vogel B, De Geest S, Fierz K, et al. Dementia care worker stress associations with unit type, resident, and work environment characteristics: A cross-sectional secondary data analysis of the Swiss Nursing Homes Human Resources Project (SHURP). International psychogeriatrics. 2017;29(3):441-454. | 1 |
| 34 | Weyerer S, Schäufele M, Hendlmeier I. Evaluation of special and traditional dementia care in nursing homes: Results from a cross-sectional study in Germany. International journal of geriatric psychiatry. 2010;25(11):1159-1167. | 1 |
| 35 | Willemse BM, Depla MFIA, Smit D, et al. The relationship between small-scale nursing home care for people with dementia and staff’s perceived job characteristics. International psychogeriatrics. 2014;26(5):805-816. | 2 |
| 36 | Zuidema SU, de Jonghe JFM, Verhey FRJ, et al. Psychotropic drug prescription in nursing home patients with dementia: Influence of environmental correlates and staff distress on physicians' prescription behavior. International psychogeriatrics. 2011;23(10):1632-1639. | 2 |
| 37 | Zwijsen SA, Gerritsen DL, Eefsting JA, et al. Coming to grips with challenging behaviour: A cluster randomised controlled trial on the effects of a new care programme for challenging behaviour on burnout, job satisfaction and job demands of care staff on dementia special care units. International journal of nursing studies. 2015;52(1):68-74. | 2 |

Table S 3: List of excluded studies after fulltext screening

Reasons for exclusion: 1= Intervention not clearly described/ no focus on an intervention, 2=Outcomes are not focused on residents/ no outcome evaluation, 3=Intervention does not fit into the logic model, 4= Setting is not nursing home or deviates from the German system
