# Supplementary material for: Context, mechanisms and outcomes of dementia special care units: An initial programme theory based on realist methodology
Source: PLoS One. 2021 Nov 16;16(11):e0259496. doi: 10.1371/journal.pone.0259496 (PMC8594822; doi:10.1371/journal.pone.0259496)
Supplement: S4 Table — (DOCX) [file pone.0259496.s005.docx]

|  | References excluded after quality appraisal because of low relevance |
| --- | --- |
| 1 | Kok, J. S., Berg, I. J., Blankevoort, G. C. G., & Scherder, E. J. A. (2017). Rest-activity rhythms in small scale homelike care and traditional care for residents with dementia. BMC Geriatr, 17(1), 137. doi:10.1186/s12877-017-0525-1 |
| 2 | Kok, J. S., van Heuvelen, M. J., Berg, I. J., & Scherder, E. J. (2016). Small scale homelike special care units and traditional special care units: effects on cognition in dementia; a longitudinal controlled intervention study. BMC Geriatr, 16, 47. doi:10.1186/s12877-016-0222-5 |
| 3 | Palm, R., Trutschel, D., Simon, M., Bartholomeyczik, S., & Holle, B. (2016). Differences in Case Conferences in Dementia Specific vs Traditional Care Units in German Nursing Homes: Results from a Cross-Sectional Study. J Am Med Dir Assoc, 17(1), 91.e99-13. doi:10.1016/j.jamda.2015.08.018 |
| 4 | Saidlitz, P., Sourdet, S., Voisin, T., & Vellas, B. (2017). Management of behavioural symptoms of dementia in a specialized unit care. Psychogeriatrics, 17(2), 81-88. doi:10.1111/psyg.12193 |
| 5 | Selbæk, G., Kirkevold, Ø., & Engedal, K. (2008). Psychiatric and behavioural symptoms and the use of psychotropic medication in special care units and regular units in Norwegian nursing homes. Scand J Caring Sci, 22(4), 568-573. doi:10.1111/j.1471-6712.2007.00576.x |
| 6 | Verbeek, H., Zwakhalen, S. M., van Rossum, E., Ambergen, T., Kempen, G. I., & Hamers, J. P. (2010). Small-scale, homelike facilities versus regular psychogeriatric nursing home wards: a cross-sectional study into residents' characteristics. BMC Health Serv Res, 10, 30. doi:10.1186/1472-6963-10-30 |
| 7 | Zuidema, S. U., de Jonghe, J. F. M., Verhey, F. R. J., & Koopmans, R. T. C. M. (2010). Environmental correlates of neuropsychiatric symptoms in nursing home patients with dementia. Int J Geriatr Psychiatry, 25(1), 14-22. |

Table S 4: List of excluded studies after quality appraisal
