# Supplementary material for: Context, mechanisms and outcomes of dementia special care units: An initial programme theory based on realist methodology
Source: PLoS One. 2021 Nov 16;16(11):e0259496. doi: 10.1371/journal.pone.0259496 (PMC8594822; doi:10.1371/journal.pone.0259496)
Supplement: S5 Table — (DOCX) [file pone.0259496.s006.docx]

| **Long term outcomes** | **Underlying context-mechanism-(intermediate)-outcome configuration** | **Indicated empirical evidence** |
| --- | --- | --- |
| **Activities** |  |  |
| Activities sustain or improve the resident’s *independence/ autonomy* if | - residents are encouraged to be independent and take part in activities *(intermediate outcomes)*. They can be encouraged by the staff, if they know the residents’ impairments and preferences, and if they provide activities *(intervention*) according to what they know about the residents *(context).* If the staff composes small groups of residents that fit well together *(context)* then supportive group dynamics evolve that trigger activity participation *(mechanism)*. - they receive one-to-one support *(intervention)* in case residents do not fit in a group or prefer individual activity *(context).* - the activities are related to daily tasks of the residents that they were doing before they moved in the nursing home *(intervention)* and if others do not constantly correct the resident or offend him/her in case he/she is performing the activity in an unusual way or less perfect*.* Not being corrected or offended increases the willingness to being active and independent *(mechanism)*. | Interviews with Stakeholders Nr. 02, Stakeholder Nr. 10 |
| Activities sustain the residents autonomy, if | - staff is present in body *(context)*, they can see what is happening and react to happenings *(mechanism)* and the residents’ are able to express their preferences and wishes *(mechanism)*. Then the staff is able to react to these expressions and meet the needs *(intermediate outcome)*. The staff’s bodily presence is dependent on care routines, leadership directions, sufficient amount of staff and architecture *(context)*. - staff is present in mind *(context)*, they can motivate the residents *(mechanism)* to be involved in the participating process *(intermediate outcome)*. Patient participation *(intermediate outcome)* is a responsibility of care staff *(context)*. - staff has the corresponding competencies *(context)*, they are able to interpret the behavior of the residents, to communicate appropriately and deliver respectful and individual care *(mechanism)*. This will impact the residents’ ability and wish to participate *(intermediate outcome)*. - the residents are able and wish to communicate their thoughts and preferences *(context),* so that the staff can react to it *(mechanism)* and facilitate participation *(intermediate outcome).* | Helgesen 2010 [40] |
| Autonomy can be enhanced if | - the staff meet the residents at eye level and don’t disparage the residents *(mechanism)* - the staff was selected on purpose to work with residents with dementia *(context)* | Stakeholders Nr. 02, Stakeholder Nr. 10 |
| Autonomy can be undermined if | - the leader of the unit is a bad role model and does not establish a feedback-culture *(context)*, then the staff doesn’t have the possibility to improve their care *(mechanism)* and will adapt the negative attitude of the leader into their moral presence when they interact with the residents *(mechanism)*. - Staff is acting in a negative and paternalistic way when communicating with the residents *(context)*, then the residents can show fear or uncertainty *(intermediate outcome)* because their decision-making capacity is linked to the staff’s behavior *(mechanism)*. | Helgesen, 2010 [40] |
| Activities sustain the residents self-esteem and dignity *(long term outcomes)* if | - staff is well-educated, committed and often on duty *(context)* they know the residents habits, routines and previous experiences *(context)* and are able to interpret the resident’s expressions of life individually *(mechanism).* Then they can base their decisions on this knowledge and decide in the sense of the resident *(mechanism)* who agrees with the decision if he/she does not shows any resistance *(intermediate outcome)*. - staff is sufficiently available *(context)* and participation is given a priority *(mechanism).* - residents are able to communicate their wishes and decisions or if relatives are able to do this *(context)*, nurses invited the residents more often to participate in decisions about every day life *(mechanism)*. Depending on how well the resident was met by the individual nurse and how well their relationship was *(mechanism)*, the residents participates *(intermediate outcome)* - care culture supports the idea of participation and the leader is visible on the unit *(context)*. Then participation is stimulated *(mechanism).* - the effects of the residents' decisions can have negative consequences for them and other residents *(context)*, then nurses adjust the choice of decision so far *(mechanism)* because then no one can come to harm and the residents can still make meaningful decisions *(intermediate outcome)*. If consequences of decisions are serious and the decisions are considered as wrong, staff offers the resident to choose from a limited number of options *(mechanism)*. Then residents were able to decide by themselves *(intermediate outcome)*. - residents are not able to make their own decisions and staff has to decide for them *(context)* and residents do not protest against the decision *(mechanism )*it is considered as participation *(intermediate outcome)*. - nurses wait for the right moment or use diverting actions *(mechanism)* when residents show resistance against care activities that nurses decided to initiate *(mechanism)* so that the activity could be done without resistance *(intermediate outcome)* | Helgesen, 2014 [41] |
| Activities contribute to the social interaction of residents if | - recreation staff *(complex intervention)* motivates residents to take part in structured activities instead of taking a nap and if the recreation staff participates themselves in the activities *(context)* because this strengthens the relationship between the resident and the staff *(mechanism)* and both are engaged in conversations during activities *(mechanism)*. Individual preferences of the residents may also be a mechanism for social interaction *(mechanism)*. - If staff is not trained how to engage with residents with dementia in conversations *(context)*, staff will not initiate conversations with them *(intermediate outcome)*, although they are eating together *(context)*. | Abbott, 2017 [34,35] |
| **Activities & Environment (Small scale living groups, Green Care Farms)** | | |
| Green Care Farms improve the quality of life if | - the care environment provides different features that are linked to former daily living of the residents (animals, stables, garden) *(context)* that residents make use of and go outside more often *(intermediate outcome).* - residents can go outside whenever they want *(context)* and do this more often *(intermediate outcome)*. Being outside more often reduces perceived stress, physical effort and agitation *(long term outcome)* because of the sunlight and the exposure to fresh air and the nature *(mechanism)*. - staff that learns how to incorporates activities into daily care practices *(input)* and motivates residents to do so *(mechanism)*. Then residents are more engaged in domestic activities and are more physically active *(intermediate outcome).* | DeBoer 2017 [38], 2017 [39] |
| Activities may improve the quality of life domain “having something to do” if  BUT | - care units implement the concept of small-scale living to a high degree *(complex intervention)*, the residents are more involved in certain (preferred) activities *(intermediate outcome)*, because the staff is more familiar with the residents history and preferences *(mechanism)* and pick up signals from the residents more easily *(mechanism)*, they are more aware when residents are inactive for a longer time *(mechanism)*. Staff may also have better organizational skills and know better how to activate residents *(context)*. The environment offers more opportunities to be involved in small activities or conversations in shared rooms *(context);* also for the staff the environment makes it easier for them to offer small activities on a frequent basis *(context)* and do this with regard to the residents’ life history and preferences *(context)*. - job demands from care staff is low *(context)* and staff is better educated *(context)*, because residents are more often involved in activities then *(mechanism)*. - If residents with a high care dependency, challenging behavior, who are older and male *(context)* are not considered by the staff as a group that needs special attention *(mechanism)* and if they do not provide tailor made activities for this group *(mechanism),* they do not perform more activities *(intermediate outcome),* because staff does not know how to make adequate activity offers and deprioritizes activities *(mechanism).* | Smit, 2012 [43]  Smit, 2017 [44] |
| Small living groups do not improve the quality of life in total | - because of measurement problems - because residents with a high QoL at baseline do not respond to the intervention | Verbeek, 2010 [45]  Kok, 2018 [42] |
| Small living groups improve the quality of life domain “having something to do” if | - other components like the attitude of the staff *(mechanism)* and the medical care *(complex intervention)* are supporting good dementia care. It is assumed that a small living unit is beneficiary only for a certain group of residents. | Verbeek, 2010 [45] |
| Small living groups may increase the feeling of homeliness (as a domain of quality of life) if | - facilities have features that are archetypal (e.g. kitchen, living room, separate bedrooms, entrance), a small group (max. 7 residents) and the opportunity to bring own furniture (both in private and shared rooms) and if the facilities provides freedom in everyday life (open and easy accessible spaces, own bedroom to retire) *(complex intervention component)* - social and organizational factors provide privacy *(mechanism)* - a limited number of residents live in the care unit *(context)*, this improves the opportunity to build a personal relationship with the staff and among the residents *(mechanism)* because the residents more often see familiar faces *(mechanism)* which increases recognition *(intermediate outcome).* - family caregivers can get involved in daily life and care *(complex intervention)*. This encourages them to exercise and maintain their (former) family roles *(mechanism)*. | Verbeek, 2012 [46] |
| Small living groups do not improve autonomy *(long term outcome)* if | - not sufficient staff or facilities are available that support activities *(context)*. - the staff has too many tasks *(context)* and their encouragement to engage residents in activities is low *(mechanism).* | Verbeek, 2012 [46] |
| Small living groups may improve autonomy *(long term outcome)* if | - residents have the choice to participate in everyday activities *(intermediate outcome*). Having the choice to participate *(intermediate outcome*), increased residents autonomy (*long term outcome*). - staff encouraged residents to participate in daily activities referring to the resident’s individual wishes, needs, strengths and capacities *(mechanism)*. This enabled the residents to continue their own lifestyle *(intermediate outcome)*. - nurses are autonomous in deciding how to plan the day so that they can orient on residents needs and preferences and not on a fixed day structure *(mechanism).* Then care is perceived as more tailor-made *(intermediate outcome).* Management facilitates this process of autonomy by empowering nurses to focus on the needs of residents rather than on the performance of scheduled tasks *(mechanism)*. | Verbeek, 2012 [46] |
| Small living groups do not improve the behavior BUT if | - the institutional policy and the staff’s attitude are more resident-oriented emphasizing individualized and psychosocial care *(context)* so that less physical restraints and psychotropic drugs are used *(intermediate outcome*). - staff perceives aberrant motor behavior not as agitation *(mechanism)* and physically non-aggressive behavior as normal acting of the residents or a result of resident’s need for stimulation *(mechanism)* because of their person-centered attitude towards care *(mechanism)*. In result, less physical restraints and psychotropic drugs are used *(intermediate outcome*). | Verbeek, 2010 [45], 2014 [47] |
| Small-living groups may improve the behavior (more social engagement, less anxiety) if | - the environment of the care unit acts as a stimulator *(mechanism).* | Verbeek, 2014 [47]  Kok, 2018 [42] |
| **Behavior management** | | |
| Extreme behavioral symptoms are avoided or stabilized |  | Stakeholder Nr. 2 |
| Behavioral symptoms are not repressed | - because they are accepted as part of the residents way of communicating and understood as autonomy *(mechanism)* | Stakeholder Nr. 2 |
| Neuropsychiatric symptoms/ Challenging behavior is reduced, if | - staff is educated in theoretical models that explain challenging behavior and they learn how to use assessment instruments to describe the behavior of the residents *(input)*, their awareness is being raised especially regarding symptoms that were previously overlooked (e.g. depression) *(mechanism)*. The multidisciplinary exchange *(complex intervention)* facilitates the thinking of alternatives to prescribing psychotropic drugs *(mechanism)* which leads to a change in prescribing behavior and lower prescription rates *(intermediate outcome)*. - behavior assessments are provided for residents with severe dementia *(context)*, the staff is more attentive to certain behavior symptoms in this group *(mechanism)* which leads to a decrease of the symptoms *(long term outcomes)*. - implementation of all intervention components succeeded well *(intermediate outcome)*. This may not the case if staff fluctuation is high *(context)* or if staff in leading positions left the unit *(context)*, because then the attention to the program is dropped *(mechanism)*. High workload *(context)* is also considered as a barrier to implementation *(intermediate outcome)* because something “extra” can not be handled and is skipped *(mechanism).* Having experiences with implementing new programs *(context)* was considered as a barrier for implementation *(intermediate outcome)*, because motivation was lower and skepticism higher then, especially if formerly implemented care programs did not improved daily care *(mechanism)*. When nursing homes are involved in more than one project *(context)*, this interfered with the implementation *(intermediate outcome)*. - “Key persons” function as team champions *(context)* in supporting the use of the care program by encouraging the staff to fill out the forms *(mechanism)*, implementation will be more successful *(intermediate outcome)*. | Zwijsen 2014 [48], Zwijsen 2014 [49] |
| Neuropsychiatric symptoms may not be reduced, if | - working schedules of different disciplines were not congruent *(context)* and interdisciplinary meeting were not held *(intermediate outcome)*, staff were disencouraged *(mechanism)* and did not filled out the forms of the program *(intermediate outcome)*. - organizational structure changes *(context),* this causes turmoil *(mechanism)* and interferes with the implementation of the care program *(intermediate outcome)*. | Zwijsen, 2014 [48] |
| **Family and public involvement** | | |
|  | - If relatives were caregivers themselves before the nursing home admission *(context)* they value the work of the nursing home staff to a high amount *(mechanism)*. Staff that feels valued, expresses positive feelings more often and is enjoying work more *(mechanism)*. This in turn affects the quality of their work *(intermediate outcome)*. - If relatives are in a close relationship *(context)*, they are willing to participate in the activities in the nursing home *(mechanism)*. Hence, they are active in organizing and performing activities *(intermediate outcome).* | Interviews with Stakeholder Nr 3, Stakeholder Nr. 4 |

Table S5: CMO-configurations developed from included studies and interviews
