# Supplementary material for: Context, mechanisms and outcomes of dementia special care units: An initial programme theory based on realist methodology
Source: PLoS One. 2021 Nov 16;16(11):e0259496. doi: 10.1371/journal.pone.0259496 (PMC8594822; doi:10.1371/journal.pone.0259496)
Supplement: S1 File — (DOCX) [file pone.0259496.s007.docx]

**BeStaDem_Realist Review Appraisal Form**

| **Document** |
| --- |
| **Title**: |
| **First author, year**: |
| **Companion papers** (multiple papers on the same study, if any): |
| Brief description of the research/report: |
| **Aim**:  **Methods**:  **Results**: |

| **Appraisal of relevance** (to be completed at the end):  Can the document contribute to the theory building? | | | |
| --- | --- | --- | --- |
| **€** high | **€** moderate | **€** low | **€** exclude |
| Notes: | | | |
|  | | | |

| **Appraisal of rigour** (to be completed at the end):  Is the method used to generate the relevant data credible and trustworthy? | | |
| --- | --- | --- |
| **€** high | **€** moderate | **€** low |
| Notes: | | |
|  | | |

| **Complex interventions provided in DSCU**  Which complex interventions are referred to in the document? |
| --- |
| 1. Activities 2. Family and public involvement 3. Behavior management 4. Staff education 5. Environment 6. Multiple |
|  |

| **Intermediate and long term Outcomes**  Which outcomes are referred to in the document? (intended or unintended; proximal, intermediate or final) | |
| --- | --- |
| 1. Activity involvement 2. Social integration 3. Less psychotropic drug/ restraint use 4. Improved case management | 1. Quality of life 2. Behavior 3. Autonomy |
|  | |

| **Linkage between C, M and O**  What is the C-M-O-configuration in the data?  *Context*: physical/social environment (e.g. cultural norms, financial conditions); pre-existing; triggers (enables/disenables) a mechanism  *Mechanism*: generative, underlying force/social behavior that leads to an outcome (sensitive to change in context); explanation why outcomes or barriers to outcome were expected  *Outcome*: intended or unintended |
| --- |
| Describe the connection(s) between the CMOs: How are the linkages between C, M and O? |
|  |

**Appraisal of relevance: Definition of the categories**

HIGH:
This category is for papers that have high relevance to this realist synthesis. This means that the framing of the research, the research questions or the outcomes produced are highly matched to the review questions, there is a rich description of the context, mechanisms and outcomes and especially explanations about the linkage between them that can greatly advance the theoretical output of the review. The paper is a ‘key informant’.

MODERATE:
This category is for documents that show a ‘moderately’ relevant contribution to the development of the program theory. This may mean that the article reports only about a few linkages between C, M and O or describes linkages between them even if there is no relevant empirical data from the paper to populate the CMO-configurations, or has a few areas that are of interest even if it is not entirely clear whether they will be used in the synthesis.

LOW:
This category is for research that has met the selection criteria in terms of relevance to the review questions but is relatively thin on the description of context and mechanism and their linkages to the outcomes described in the paper. It is not placed in the exclusion category because it contains at least one idea or statement about the linkage of context, mechanisms and outcome or about conceptualizing outcomes that can be used for refining the theory and building a CMO configuration.

EXCLUDE:
Studies with a low to moderate quality are excluded.

(informed by documents of Realist Summer School 2017, Jagosh et al.)

**Appraisal of rigour**

Criteria suggested by Dixon‐Woods et al. (2005: 28) to appraise every empirical paper, regardless of its study design (aim: to identify “fatally flawed” papers)

| Are the *aims and objectives* of the research clearly stated? | **€** Yes | **€** No | Notes |
| --- | --- | --- | --- |
| Is the research *design* *clearly specified* and *appropriate* for the aims and objectives of the research? | **€** Yes | **€** No | Notes |
| Do the researchers provide a clear account of the process by which their *findings were produced*? | **€** Yes | **€** No | Notes |
| Do the researchers display *enough data* to support their interpretations and conclusions? | **€** Yes | **€** No | Notes |
| Is the *method of analysis* *appropriate* and adequately *explicated*? | **€** Yes | **€** No | Notes |

Dixon‐Woods M, Kirk MD, Agarwal MS, et al. Vulnerable groups and access to health care: a critical interpretive review. In: National Coordinating Centre NHS Service Delivery Organisation R & D (NCCSDO), ed., 2005:496.
